# Unpacking Community-Based Youth Mentoring Relationships: An Integrative Review

**DOI:** 10.3390/ijerph18115666

**Published:** 2021-05-25

**Authors:** Limor Goldner, Adar Ben-Eliyahu

**Affiliations:** 1 The Emili Sagol Creative Arts Research Center, Faculty of Welfare and Health Sciences, School of Creative Arts Therapies, University of Haifa, Haifa 3498838, Israel; 2 Department of Human Development and Counseling, Faculty of Education, University of Haifa, Haifa 3498838, Israel; adarbe@edu.haifa.ac.il

**Keywords:** youth mentoring, mentoring relationship, mentors, mentees, formal mentoring, community-based mentoring

## Abstract

Formal community-based youth mentoring relationships (CBM) are a popular form of intervention worldwide in which caring, non-parental adult figures are matched with at-risk children (i.e., children who experience an intense and/or chronic risk factor, or a combination of risk factors in personal, environmental and/or relational domains that prevent them from pursuing and fulfilling their potential) to promote development and health. Common models suggest that a close mentoring relationship is needed for the success of the intervention. However, it remains unclear which key relational processes and variables promote relationship quality to generate the most significant benefits. Using the PRISMA framework, 123 articles were identified as relevant for this review which explores the state of the literature on CBM relationships describing the main findings regarding the characteristics of the relationship and the mediating and moderating variables. An essential ingredient that consistently emerged for generating mentoring outcomes is characterized by feelings of support, sensitivity, and trust and accompanied by a purposeful approach to shaping the goals of the relationship. A balanced approach comprised of recreational, emotional, and catalyzing aspects has been reported as essential for mentoring success. Mentors’ positive attitudes toward underprivileged youth, maturity in terms of age and experience are essential in forging positive relationships. Mentees who have better relational histories and more positive personality traits exhibited higher relationship quality. However, data imply the possibility of addressing mentees from moderate risk status. Preliminary evidence on thriving as a mediating variable was found. Program practices, such as training, parental involvement, and matching based on perceived similarities and similar interests, emerged as important factors. Generating many research suggestions, the review identifies research questions and uncharted territories that require inquiry.

## 1. Unpacking Community-Based Youth Mentoring Relationships: An Integrative Review

Youth mentoring, defined as a special caring dyadic relationship between non-parental adults and their mentees that aims to promote young people’s personal and professional development, has been acknowledged as a vital asset for youth development [1,2]. Under optimal conditions, this relationship would evolve naturally from the young person’s social network and would include extended family members and informal social networks (e.g., neighbor, coach) or more formal figures (e.g., teacher, counselor; [3]). Data from the U.S. indicate that approximately 50–80% of American children and adolescents report having a meaningful relationship with non-parental adults (e.g., [4,5]), which is associate with a variety of positive short- and long-term outcomes in the behavioral, socioemotional, academic, and vocational domains (see meta-analysis by Van Dam et al. [6]). On the other hand, approximately one-third of all teens in the United States, mostly from the lowest socioeconomic quartile, report never having experienced a mentoring relationship [7,8]. More disconcerting, a troubling subgroup, making up approximately 10% of the sample, stated that there were no adults in their life from whom they could seek help [9].

To provide young people with a resource for development and social integration that is lacking in their natural social web, organized mentoring programs have promoted formal mentoring relationships, either in the community or in the school setting [10]. In community-based mentoring (CBM), mentoring programs match an adult volunteer with a child or teen referred to the program. They spend one-on-one time in neighborhood settings regularly over a determined period (e.g., one year) to facilitate this young person’s developmental goals in the socioemotional and the academic domains, employing a wide range of relational, recreational, goal-oriented/instrumental activities [11].

Approximately 4.5 million young people are involved in community-based-mentoring relationships in the U.S. alone [4]. Meta-analyses of quasi-experimental and experimental evaluations confirm the potential of CBM relationships, involving high-risk young people to progress in the socioemotional, behavioral, and academic domains [12,13,14] and in reducing delinquent and aggressive behavior [15,16]. Nevertheless, these meta-analyses have only identified small overall positive effects, with standardized mean difference effect sizes (*d*), from 0.18 to 0.21 in DuBois et al. [12,13] from 0.11 to 0.29 in Tolan et al. [16], and 0.21 in Raposa et al. [17].

Many widely used models of youth mentoring adopt the underlying theoretical assumption that a close, interpersonal relationship between a mentor and mentee is critical to ensure the success of the mentoring relationship [18]. In particular, several developmental-clinical psychological conceptualizations based on theories of attachment [19,20], social support [21,22], and social learning [20] are employed as theoretical frameworks to analyze the potential significance of formal mentoring in the lives of young people. These theories argue that the character development linked to the mentoring relationship derives mainly from primary prevention, a conclusion derived from applying a “deficit” approach that focuses on the mentees’ difficulties [8,23].

Recently, the literature has begun to adopt a more positive developmental theory-based approach that shifts away from viewing young people as deficient and needing treatment to a more positive empowering framework [8,23]. Rejecting earlier conceptualizations of young people as disadvantaged and “fundamentally flawed,” this framework sees young people as congenitally competent, talented, and eager for positive development and health [23]. This approach implies that mentoring goals and practices should be ‘thriving-oriented’ rather than deficit-oriented [24]. One of the key theories concerning mentees’ thriving is Positive Youth Development (PYD; [25]).

However, despite the recent surge in mentoring literature, considerable gaps remain in our understanding of which are the critical relational processes that generate lasting benefits for young people in CBM [26]. Attaining a deeper understanding of the dynamics of mentoring is nevertheless crucial, given that the estimates of average annual cost per child or adolescent in CBM programs in the U.S. stand at $1647, and the cost per new enrollee in a mentoring program is $3301, assuming it lasts 19 months [27] Thus, in order to gain a broad, updated perspective on the qualities of the relationship associated with mentoring outcomes (distal outcomes), as well as the factors promoting the quality of the relationship and its longevity (proximal), the current integrative review was designed to summarize and discuss the critical quantitative and qualitative findings from the past 20 years on one-on-one, face-to-face CBM relationships. These years have seen a substantial enhancement in the published studies’ sophisticated methodology and validity. Thus, reviewing studies published during this period provides a current window on this topic. Specifically, we reviewed the following research questions (RQ): (RQ1) What are the main findings related to the characteristics of the relationship associated with mentoring satisfaction, length and outcomes?; (RQ2) What are the current measurement approaches and measures utilized to assess the relationship?; (RQ3) What are the mediating processes that have been found to influence relationship quality?; and (RQ4) What are the moderating variables that moderate the quality, duration, and satisfaction of the relationship? 

This overview extends previous efforts (e.g., [10,26,28]), which were more limited in scope, typically focusing on specific publications or research to support the conceptual or theoretical literature, but did not conduct a methodical search of literature or a broad systematic review. For instance, Deutsch and Spencer [26] dealt primarily with measurement issues in CBM, school-based mentoring (SBM), and natural mentoring. Schwartz et al. [29] summarized the effects of mentoring relationships on self-esteem and the processes through which these relationships exert such effects in CBM, SBM, and natural mentoring. The review by Rhodes and DuBois [10] surveyed mentoring best practices across a broad spectrum of youth-serving settings in an effort to promote better alignment of research and practice. Stewart and Openshaw [28] addressed the difficulties of defining the term “mentor” and mentoring benefits. The notable meta-analyses by DuBois et al. [13], and that of Raposa et al. [17] were primarily aimed at identifying the effectiveness of one-to-one, group and e-mentoring and its moderators, using quantitative randomized control trial (RCT) studies. These studies addressed the effectiveness of the intervention as their focus, highlighting the quality of the relationship as a potential moderator for the effectiveness of the interventions, comparable with other possible moderators (e.g., program and organization characteristics, mentees’ and mentors’ characteristics, and the family and community context). In most cases, these studies referred to the relationship employing dichotomous variables (e.g., format, orientation, amount of contact, duration), and, as conventional practice in meta-analyses, they eschewed qualitative findings that could shed light on the experience of the relationship. The current review is, therefore, a natural, integrative extension of these works, concentrating directly and thoroughly on identifying the qualities of the relationship associated with mentoring outcomes and the factors that promote or inhibit its quality.

## 2. Method

### 2.1. Search Strategy

A comprehensive search of the literature published between January 2000 and April 2020 was conducted by the first author. Both computer-based and manual search methods were used to identify pertinent studies. The computerized databases utilized were PsychINFO, ERIC, PsycNET, PubMed, Scopus, and Web of Science. The search of each computerized database included the following terms and combinations of terms: “youth mentoring relationship,” “youth mentoring relationship + community-based mentoring,” “youth mentoring relationship + mentoring length,” “youth mentoring relationship + mentoring duration,” “youth mentoring relationship + relationship quality,” “youth mentoring relationship + per/early-match termination/early closure,” “youth mentoring relationship + mentors’/mentees’/protégés’ characteristics,” “youth mentoring relationship + relational/relationship characteristics,” and “youth mentoring relationship + culture/context/gender/sex/race and ethnicity,” These searches yielded 1308 full-text peer-reviewed articles, dissertations, research and technical reports, and book chapters written in English.

### 2.2. Study Selection Procedure and Inclusion Criteria

The first author conducted the screening. Dilemmas were resolved through discussions with the second author. Both researchers are experts in the field of youth mentoring. Publications were included if they were qualitative or quantitative empirical studies, research reports, or meta-analyses and review articles dealing with formal one-to-one face-to-face community-based mentoring. In some cases, they were site-based. The mentor relationship could be either short-term (<12 months) or long-term (≥12 months), as well as applying multi-component programs, such as skills-group components. For the current review, we consider mentoring as comprising a one-on-one interaction between a non-parental adult figure and a specific younger person (aged 6–25) to promote positive outcomes for the latter through the relationship. 

In the first round of screening, 692 publications addressing natural mentoring, professional, peer/cross-age mentoring, academic mentoring, work/vocational mentoring, or those concentrating solely on school-based or group mentoring were excluded. In addition, specific mentoring interventions, such as mathematics, sports, and health interventions, were also excluded. We chose to include 15 studies that addressed both CBM and SBM and six multi-component programs, such as mentoring interventions that also included skills groups. This decision was taken out of the desire not to exclude pivotal meta-analyses in the field of youth mentoring (see, for example, meta-analyses 25, 38, 41, and 129) and given the scant findings that address the various aspects of the mentoring relationship. Next, 208 duplicate items were excluded. Subsequently, the studies underwent a second round of screening, which excluded 272 technical reports, toolkits and manuals, conceptual work, editorials and commentaries, book chapters, as well as articles focusing solely on mentoring effectiveness that did not treat the quality of the relationship, relationship duration, relationship intensity, types of provisions, activities, structure, or satisfaction. 

**Figure 1:**
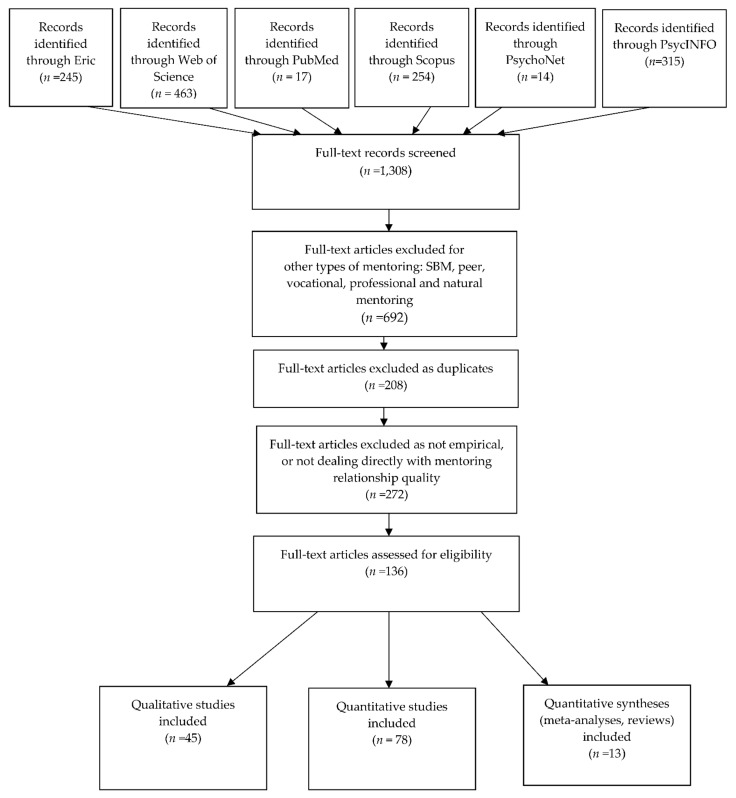
Flow chart of the methodology applied to screen records.

Similarly, we excluded studies addressing young people’s social support or social capital in general or studies concentrating only on young adults. These screening stages yielded 136 empirical studies (45 qualitative, 78 quantitative, and 13 reviews and meta-analysis articles). The selection flow, presented in Figure 1, was based on the PRISMA (2020) [30] procedure for transparent reporting of systematic reviews and meta-analysis. Note, however, that deviating from the PRISMA process, during the screening process, the exclusion phase of studies addressing other types of mentoring was performed before excluding of duplicated studies. 

**Figure 2:**
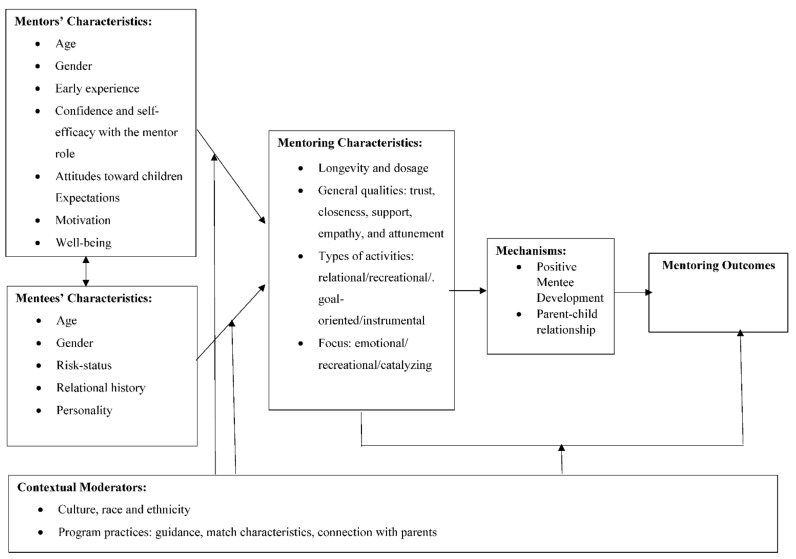
The determinants and mechanisms of mentoring relationship quality.

Fifty (37%) of the 136 studies were conducted in the Big Brother Big Sister (BBBS) program setting. Of the identified studies, 12 studies were gender-based, concentrating on studying boys’ or girls’ experiences. Ten articles referred to both adolescents and young adults. Thirteen studies addressed special mentored populations (two from the juvenile corrections system, two mental health clients, three concerning foster care, three residential care, five concerning immigrants and refugees, one concerning homeless mentees, and one cystic fibrosis mentees). The aim of the study, the sample characteristics, the program characteristics, country, study design, main analyses, and main findings, all coded by the first author and the Ph.D. student, are presented in Supplementary Tables S1–S3. Thirty-seven studies (27%) were coded jointly, whereas the remainder were coded separately. Dilemmas involving coding judgments were resolved through discussion by the two coders. To achieve deeper insights into the mentoring process, aside from the identified studies, we have supplemented and integrated theoretical writings and book chapters throughout the review (Figure 2).

## 3. Results

This section is structured according to the four posited research questions. First, the primary findings concerning the critical characteristics of the mentoring relationship are reviewed as they relate to mentoring outcomes. The second section discusses methodological and measurement approaches. Findings on the mediating processes are discussed in the third section. We conclude with the precursors and moderating variables reported to influence relationship quality. Aspects of mentor and mentee, as well as gender, culture, program practices, and match characteristics associated with the duration, quality, and satisfaction of the relationship, are cited. The conclusion proposes directions and topics for future research. Throughout the review, the state of the art is summarized and subjected to a critical lens (see Figure 2 and Table 1 for the study questions and the organization of the review). Note, however, that the varied nature of CBM (e.g., long-term versus time-limited; one-to-one versus group mentoring; goal-directed versus relationship-focused) and the populations served (e.g., youth involved in the juvenile justice system, foster youth, at-risk youth) makes this type of review challenging to report. Indeed, programs place varied emphasis on the quality of the relationship depending on their theory of change, and, as a result, the quality of the relationship may or may not be critical to positive youth outcomes.

### 3.1. RQ1: Relationship Characteristics

In the past twenty years, studies have taken pains to identify the critical ingredients of relationships that contribute to mentoring success, duration, strength, and satisfaction in terms of the length of the relationship, its emotional tenor, and its associated activities, techniques, and practices.

*Mentoring length and dosage*. The mentoring relationship is an evolving and dynamic entity that includes a series of stages that are often classified as contemplation, initiation, growth and maintenance, decline and dissolution, and redefinition [31]. The extent to which young people benefit from the mentoring experience depends on the extent to which the dyad has reached the growth and maintenance phase [31]. Therefore, the duration of the mentoring dyad and its consistency have been identified as critical features [12,32].

Understandably, most mentoring relationships are short-term affairs and are limited in their time structure. However, numerous quantitative studies, mainly conducted in the context of the BBBS programs, and as such are open-ended with no pre-specified end date, have shown that the quality of the relationship, the duration of the mentoring, and its intensity are associated with levels of the mentees’ adjustment at the conclusion of the intervention. Data from random assignment studies comparing mentees and a parallel control group on a waiting list for BBBS programs in the U.S. found that the effects of mentoring on adolescent outcomes became progressively stronger with match length [33]. Specifically, young people who were in matches that lasted more than 12 months exhibited increases in their self-worth, perceived social acceptance, perceived scholastic competence, parental relationship quality, school value, and a decrease in both drug and alcohol use. In contrast, mentoring relationships that ended prematurely, at between three and six months, despite the open-ended intentions of the mentoring relationships, resulted in decreased perceived self-worth and scholastic competence [33].

Similarly, mentorship duration (M = 14.51 months) was significantly associated with youth greater competence in BBBSA [34]. A recent analysis of BBBS CBM programs in Canada documented a drop in emotional and behavioral problems in long-lasting matches. The results showed that mentored young people, especially those in mentoring relationships lasting 12 months or more in an open-ended mentoring relationship (continuous or dissolved), manifested fewer behavioral problems or symptoms of depression or social anxiety than did non-mentored young people [35]. This finding is interesting, given that the average match length for BBBS America is 11.4 months [36]. Longer relationships and few or minimal match difficulties were positively correlated with higher relationship quality, as reported by parents and mentees among adolescents from 20 agencies from BBBS Canada [37] and mentees in mentoring programs in Sweden [38]. In their Swedish study, Larsson et al. [39] demonstrated the importance of sufficient duration in a study conducted in a mentoring program that lasts for at least one year. They reported how females with mental illness advanced from feelings of embarrassment and nervousness to authentic, undemanding, and non-hierarchical relationships. 

Furthermore, mentors’ perceptions [40] and mentees’ perceptions [41,42] regarding the general quality of the relationship predicted mentoring longevity in U.S. and Canadian BBBS programs. Notably, the findings regarding the contribution of mentoring duration in meta-analyses are mixed. Nevertheless, attention should be paid to the array of types and formats that these studies have explored. For example, DuBois et al.’s [13] meta-analysis encompassed 73 short and long-term programs, including peer-mentoring, CBM, and SBM, mentoring in formats of one-on-one, group mentoring, and e-mentoring, indicating extended positive effects in programs with a relatively pre-defined brief duration (i.e., less than six months). However, DuBois et al.’s [12] meta-analysis of 55 studies conducted in the context of CBM, SBM, and vocational mentoring did not find an observed effect of mentoring length. Similarly, a recent meta-analysis of 70 CBM and SBM mentoring programs [17] and a meta-analysis composed of five studies on mentoring programs among youth with externalizing and internalizing behavioral problems [43] did not find an observed effect based on program length. However, programs with expectations for longer match durations produced smaller effect sizes [17].

Dosage of the mentoring intervention was also suggested as an important determinant of mentoring relationship quality and outcomes. Nevertheless, here, too, the findings regarding its effect on the mentoring outcomes are mixed. For instance, DuBois et al.’s meta-analyses [12,13] indicated that the average frequency of contact did not serve as a significant moderator of effect size. However, analyzing data drawn from a national survey of mentoring programs for mentored youth (referred from the juvenile justice system in the U.S.) indicated positive associations between frequent interactions and meeting length with the program staff’s success ratings [44]. Moreover, the amount of time mentors and mentees spent together predicted increases in academic outcomes and declines in drug use in BBBS America [45]. The number of mentoring visits attended was associated with mentoring relationship quality in mentors and mentees’ reports in the Campus Connection program, which is a short 12-week program [46].

*Quality of the relationship: Core ingredients*. Beyond issues of time, models evaluating mentoring success point to the relationship’s quality as the primary vehicle of change [8]. These models primarily assess the general tone of the relationship as it associates with positive and negative aspects of the relationship, such as support, closeness, help, and trust versus disappointment, dissatisfaction, and conflict. Studies conducted in various short-term and long-term mentoring programs, with determined and undetermined termination points, showed that mentors’ rating of how much support they provide to their mentees was related to a decrease in mentees’ aggressive behavior [47,48]. Mentors’ rated support was also related to an increase in empathy, cooperation, self-control, assertiveness [49], social self-efficacy, and sense of community [50]. Mentees’ rated feelings of trust and closeness with their mentors were positively associated with an increase in social support and family bonding, scholastic competence, feelings of self-worth [18,51,52,53,54], self-regulation [55], hope, self-esteem, self-efficacy, academic pursuits [56], active coping skills [57], general mental health and career efficacy [58], academic outcomes [45], and future-planning style and career goal setting [59]. Similarly, mentees’ perceived high support from their mentors and low conflict within the mentoring relationship predicted a decrease in externalizing problems in a long-term mentoring program aimed at reducing aggressive behaviors [47]. Positive mentoring relationship reported by the mentees and assessed by aspects of happiness and understanding negatively predicted mentees’ marginalization in a cross-sectional study conducted in a two-year mentoring program in Rwanda [60].

Mentors’ and mentees’ perceived support was related to the duration of the relationship in BBBS of America [42]. Mentees’ perceptions of support and help from their mentors, happiness, and mentor satisfaction were positively associated with the mentees’ academic outcomes (e.g., liking school, scholastic efficacy, grades, education plans), social outcomes (e.g., social acceptance, parental trust, social support from parents, siblings, and other adults), and emotional outcomes (e.g., hope) in BBBS in Ireland [61]. Mentees with attuned mentors (i.e., exhibiting an ongoing capacity to identify and flexibly meet mentees’ needs; [62], who participated in a 12-week time-limited mentoring program, reported greater value for school, academic self-efficacy, and truancy, as compared with mentees with poorly attuned mentors [63]. Perceived mentors’ help and mentees’ dissatisfaction, the latter feeling hurt and betrayed, accounted for poor mentee outcomes in BBBS [18]. Perceived mentees’ asymmetrical relationships with their mentors predicted low levels of high-risk mentees’ perceived mentoring contribution to their social and academic functioning in an 18-month program in China [64]. Finally, mentors who rated the mentoring relationship as supportive tended to experience increased openness, conscientiousness, and agreeableness and less attachment avoidance at the end of the intervention [65].

Nevertheless, a critical limitation of some of these studies is that few included control groups or used reports from the same informant (usually the mentees) on the quality of the relationship or the level of functioning, thus possibly creating shared method variance (e.g., [52,53,54,61,64]).

*Types of activities*. Beyond detecting general feelings of support and closeness, many studies have sought to unpack how specific objectives, actions, and interactions in which mentors and young people are engaged impact the perceived supportiveness of the relationship and its benefits [66,67,68]. Indeed, studies comparing the contribution of relational and recreational focus (i.e., interactions concentrating on relationship building and strengthening to promote emotional well-being) and engagement with a more goal-oriented and instrumental approach (i.e., interactions that target specific behavioral goals and skills using structured activities) [68,69] have proved inconclusive. Whereas several studies reported the benefits of the mentor relationship focusing on relational goals and interactions, others pointed to the advantages of a more goal-oriented and instrumental approach.

For instance, discussions about family and friends were related to the quality of the mentor relationship as perceived by the mentors, whereas discussions about school and future plans were significant predictors of relationship quality according to the mentees’ reports [70]. Mentors’ emotional support, rather than mentors’ instrumental support, predicted the quality of the relationship as perceived by the mentees [37]. More CBM mentors reported feelings of closeness toward their mentees than SBM mentors in BBBS of America [71]. A higher frequency of recreational activities reinforced the positive association between mentees’ perceptions of received support and relationship quality, whereas a higher frequency of tutoring activities decreased this association [72]. The use of digital media between mentors and mentees was associated with higher relationship quality and duration, as reported by mentors [73]. 

No benefit was found for programs that adopted a primary emphasis on instrumental aims or when there was a focus on providing specific skills training within a structured framework [12,13]. However, these studies showed that mentoring programs in which mentors embraced a more goal-directed interaction, using “teaching,” “coaching (i.e., instructing and training),” or “advocacy” (i.e., teaching youth to promote interests and rights) techniques [13] and applied structured mentor-mentees activities [12] led to more substantive effects than did programs not facilitating this role. A similar tendency was recently reported by Christensen et al. [74], who re-analyzed Raposa et al.’s [17] database. The study revealed that the overall effect size of goal-oriented programs was more than double that of non-specific relational programs in terms of academic, psychological, and social outcomes. 

Coaching contributed to skill acquisition and knowledge learning in a long-term mentoring program with Malaysian youth [75]. A non-directed staff approach to supporting mentors predicted lower mentee-reported relationship qualities in BBBS of America [76]. Mentees in BBBS of America who characterized their relationships in terms of a “moderate” level of activities, structure, and setting limits and a lower level of support reported more numerous benefits, including less alienation from parents, fewer conflicts and inequality with friends, and an improved sense of self-worth and school competence relative to controls. Surprisingly, mentees who experienced higher levels of support and lower levels of activity and structure reported an increase in parental alienation and did not report any benefit from the intervention [77]. The researchers suggested that engaging in recreational, relational, and instrumental activities in a relatively structured relationship setting may be interpreted by the mentees as a proxy for higher levels of mentors’ emotional investment and commitment to the relationship [77]. Finally, integrating structured group activities within the relationship was associated with mentors’ satisfaction in a short-term therapeutic mentoring program, as reported in a qualitative study [78].

Overall, these findings may imply that prioritizing a more goal-directed approach—targeting specific skills instead of concentrating on building an emotional approach—, as well as engaging in recreational and relational activities in a relatively structured relationship setting, may be more suitable for CBM mentoring programs. These findings shed new light on the objective and the course of the traditional view of the mentoring relationship, which prioritized a developmental and emotional approach rather than a goal-directed and instrumental mode [79]. Moving beyond the conceptualization of instrumental versus emotional relationships, qualitative studies have also explored the underlying relational processes that foster close mentoring relationships [80,81,82]. Three key active ingredients have been recognized: emotional, recreational, and promoting-catalyzing [82,83,84]. The emotional component, which is the most frequently engaged, includes aspects of trust, reliability, consistency, support, and authenticity in mentoring programs in all formats operating with American, European, Israeli, and Australian youth [78,80,85,86,87,88,89,90,91,92,93,94,95,96]. The emotional component also encompasses other qualities, such as listening, empathy, attunement [62,96,97,98], genuine respect [99], and sensitivity [39,94,100]. In this vein, trying to unpack the terms “empathy” and “mentors’ attunement,” researchers underscored aspects of mentors’ perspective-taking and adaptability and flexibility to youth needs [62,101]. Furthermore, Youth who described their mentoring relationship with high levels of alliance and belonging experienced high levels of empathy and acceptance on the part of their mentors [102].

The recreational component refers to connectedness, companionship, friendship, and ongoing communication [80,82,91,92,94,96,97,98,99,103,104], accompanied by collaboration, mutuality [39,86,94,95,96,103,105], and self-disclosure [106,107].

The third component of promoting-catalyzing encourages self-esteem, autonomy and self-direction, role modeling, guidance, advocacy, empowerment and challenge, future planning, and promoting motivation. Other elements of this component include skill-based career and academic support [80,82,83,85,86,88,92,93,98,108]. Yanay-Ventura and Amitay [98] further include goals, such as anger management. Expanding on this component, Brown [86] highlighted the role of critical thinking, whereas Garraway and Pistrang [107] underscored the importance of advocacy.

Conceptual writing on mentoring has emphasized mentors’ ability to move wisely and flexibly between the three dimensions, combining both a hierarchical position as an authority figure and a horizontal position as a friend [97,109]. Quantitative studies may benefit from operationalizing these three dimensions (i.e., emotional, recreational, and promotion) into measurable variables and exploring each component’s specific and shared contribution in order to better understand the mechanisms underlying mentees’ development. 

### 3.2. Mentoring Termination

Ideally, formal mentoring relationships should terminate at the conclusion of the prescribed period or when the need for the mentoring relationship has lessened [110]. Studies dealing with this issue have sought to assess termination and its impact. Specifically, findings from open-ended mentoring programs from Canada and the U.S. (mostly BBBS) indicated that 34% to half of all mentoring relationships end prematurely, with most terminating within the first few months [11,33,41,111,112,113,114]. Despite the prevalence of early-match closures, researchers have only recently begun to investigate the reasons for early termination and its adverse consequences [115,116].

Young people participating in open-ended mentoring relationships in the context of BBBS of America that terminated within three months of its initiation reported a decrease in self-worth and perceived scholastic competence [33]. Likewise, qualitative studies conducted in BBBS of America’s open-ended programs indicated that children and teens who experienced premature match closure or poorly managed terminations reported feelings of rejection, disappointment, sadness, anger, confusion, self-criticism, doubts as to positive relationships in the future, and showed less willingness to engage in subsequent mentoring opportunities [115,117]. Some programs sought to minimize the potential harm by re-matching mentees who experienced early termination. However, BBBS Canadian data comparing mentees in full matches with those who were re-matched found that youth re-matched with another mentor experienced almost no health or social benefits, implying that re-matching did not compensate for the consequences of premature termination [111].

Qualitative studies in the U.S. and Australia on mentoring programs of variable length identified several reasons for early termination, based on interviews with triads of parents, mentees, and mentors who had experienced early terminations. Their reported reasons for early termination included genuinely unforeseen changes in the mentors’ and mentees’ life circumstances, mentees’ dissatisfaction, disappointment or disinterest, mentor dissatisfaction, unrealistic expectations on the part of the mentors or the mentees, the mentors’ lack of relational skills, parents’ interference and lack of parental support, gradual dissolution with neither party investing the effort to maintain the interaction, and mentors’ and mentees’ abandonment. Weaker relationships were more likely to end as a result of mentors’ or mentees’ dissatisfaction with the relationship, or to dissolve without formal termination [116,118,119,120,121]. Mentees’ avoidance, distrust, fear of intimacy and rejection, and mentors’ overwhelming derived from the mentees’ needs and difficulties discussing the termination of the relationship were noted as reasons for early termination in a short-term mentoring program for pregnant teens [122].

A quantitative study conducted on seven mentoring programs in the U.S. on 1310 matches revealed that mentors initiated the termination in more than half the cases. Two of the most common reasons cited by mentors were lack of mentee interest or the need for a mentor [112]. A mixed-methods American study that collected data from parents, mentees, mentors, and program staff about the closure process indicated that the mentors initiated most relationship endings. In most cases, the endings were dissolved unexpectedly and unclearly, leaving parents to manage the closure with their child [121]. More quantitative studies are needed to better understand the causes and consequences of early termination of the mentoring relationship in the various formats (long, short, determined, or undetermined endpoints).

### 3.3. RQ2: Current Measurement Approaches

Relatively scant research has advanced the development and validation of measurement tools. There is a lack of consensus on what constitutes mentoring relationship quality. Whereas several researchers have focused on action-oriented processes, including the type of engagement and provided support, others have concentrated on relational processes in terms of closeness and trust [37].

In general, two approaches have been applied to assess the quality of the mentor-mentee relationship. The first employs versions of questionnaires adapted from the fields of psychotherapy, teaching, and parenting. For instance, Goldner and Mayseless [51] used the Student-Teacher Relationship Scale [123] Goldner [124] utilized the Mother-Father-Peer (MFP) Scale [125] and Chesmore et al. [57] applied a modified version of the Inventory of Parent and Peer Attachment [126]. Likewise, Cavell and Hughes [48] and Cavell and colleagues [47] employed modified versions of the Network Relationship Inventory [127], which assesses support and conflict in parents’ and peers’ relationships, as well as the Therapeutic Alliance Scale [128]. The use of these questionnaires derives from the assumption that the mentor-mentee relationship is analogous to the dynamics characterizing therapist-child or parent-child relationships because it synthesizes aspects of warmth, acceptance, and autonomy [129,130]. The advantage of this approach is that it facilitates evaluating mentoring relationship quality based on well-established theory and instruments.

The second approach aims to develop specific measures to capture the specificity of the mentoring relationship by assessing the positive and negative aspects of the relationship (e.g., [18,42,49,54]). However, the instruments used are self-reports and not always sufficiently validated or theoretically well-grounded. Indeed, most of these tools were developed using structural validity, repeatedly demonstrating that the emotional dimensions of mentoring loaded onto a single general dimension of warmth, closeness, empathy, and trust (or the absence of these dimensions), but failing to distinguish between the specific qualities of the relationship. In most cases, concurrent validity and test re-test reliability were not examined. Further work is needed to validate these tools through gathering information from multiple sources.

For example, Rhodes et al. [131] developed a 15-item mentoring relationship quality inventory completed by mentees. The questionnaire is comprises four moderately to strongly interrelated scales (Not Dissatisfied, Helped to Cope, Not Unhappy, and Trust Not Broken) to tap the positive and negative aspects of the relationship. However, data from mentees who participated in BBBS America revealed that the three-item scale (Helped to Cope) that examines positive aspects of the relationship had limited predictive power. The researchers concluded that successful mentoring relationships tended to be defined less by positive qualities and more by the absence of disappointment and negative feelings [18]. A more recent version of the scale consisting of 10 mentee-reported items (YSoR) and 14 mentor-reported items (MSoR) was developed to achieve a more balanced perspective of the relationship [42]. Again, a single factor emerged for the YSoR, assessing a general feeling of support and trust, and two factors emerged for the MSoR—the affective dimensions of the relationship and the logistic dimensions of the relationship. 

In seeking to capture the mentoring alliance beyond the absence of disappointment and negative feelings, Sale et al. [49] developed a scale that evaluates a general positive feeling in the relationship, which was tested on a sample of mentees in a community-based mentoring program in the U.S. Again, the 23-item questionnaire measures mentees’ perceptions of the presence of trust, care, support, empathy, and shared interests. Likewise, inspired by psychotherapy, Zand et al. [54] developed the Mentor-Youth Alliance Scale (MYAS). The scale comprises two five-item subscales assessing young people’s perceptions of acceptance and caring. Confirmatory factor analysis indicated a one-factor solution that assesses a broader alliance construction. Similarly, Liang et al., [132] developed a six-item scale to study growth-fostering mentoring relationships. Although the scale was designed to assess aspects of engagement, authenticity, and empowerment within the relationship (e.g., “My mentor helps me to get to know myself better”), it showed good psychometric properties for a one-factor solution. 

Several scales have been designed to assess the engagement attributes of the relationship beyond trust and support. These include the Global Mentoring Relationship Quality Scale (G-MeRQS) and the Quality of Mentoring Relationship Engagement Scale (Q-MRES) [40]. The G-MeRQS is designed to assess mentees’ general feelings of trust, warmth, happiness, and respect, using five items. The Q-MRES seeks to capture the action-oriented supportive interactions between mentors and mentees (e.g., asking to do things together, showing interest in shared activities, and asking for each other’s opinions), using 22 and 13 items for mentees and mentors, respectively. 

Future studies should develop new scales that measure the contribution of mentors’ specific behaviors and practices with the assistance of a panel of experts and weighing them against other instruments assessing relationship dimensions across other types of close interactions. This kind of examination could help determine whether the superiority of the one-factor-solution stems from a phenomenon similar to the “common factor” or the “Dodo bird verdict,” underscoring the centrality of warmth, genuineness, and empathy in psychotherapy and the relatively scant empirical evidence as to the advantages of specific techniques over others [133].

### 3.4. RQ3: Mechanisms through which the Mentoring Interaction Influences Relationship Quality and Duration

Theoretical and preliminary empirical endeavors have been made to identify processes inherent to mentoring relationships, focusing on associations between general relationship quality and mentee development. A prominent model of mentoring relationships is Rhodes et al.’s (2006) model of youth mentoring. Drawing on theories of parent-child, teacher-child, and peer relationships, the model posits that a close mentoring relationship stimulates three intertwined processes: (1) enhancement of social and emotional development; (2) improvement in cognitive functioning; and (3) promotion of positive identity development, all of which subsequently result in positive outcomes. Although [13] meta-analysis used these mechanisms as indicators of mentees’ growth, this mediation model has yet to be empirically tested.

Recent empirical efforts have posited that the five Cs (competence, confidence, connection, care and compassion, character) of Positive Youth Development and positive psychology constructs, such as optimism and hope, can serve as potential mediators between mentoring support and mentee outcomes [134]. For example, in a longitudinal study on mentees in BBBS Canada, the five Cs were found to mediate the associations between mentees’ perception of mentoring support and decreased levels of emotional and behavioral problems [35] Positive engagement with thriving activities (i.e., activities concentrating on growth mindset, strengths exploration, and goal setting and pursuit) in a mentoring program aimed to promote thriving in the context of BBBS America predicted mentee’s enhanced support for thriving from adults. This, in turn, increased mentees’ personal resources for thriving and lessened behavioral problems [11].

In a longitudinal study conducted in BBBS America [135], the parent-child relationship partially mediated the relationship between the quality of the mentoring relationship and mentees’ substance use, global self-worth, school value and attendance, and grades. The mentees’ perceived closeness with their mentor mediated the association between the mentors’ sense of efficacy at the beginning of the relationship and their perceived benefits at the end of the mentoring, as well as the prospect of relationship continuation in BBBS America [136]. These findings should be replicated in future research, along with identifying additional developmental trajectories. 

### 3.5. RQ4: Moderators Factors of Mentoring Relationships: Mentor and Mentee Characteristics, Culture, Program Practices, and Matching Criteria

*Mentor characteristics*. To maximize the potential of the mentoring relationship, researchers have sought to identify specific preexisting characteristics of mentors that are associated with the mentoring relationship quality and relationship length. These moderators include age, gender, early experience in helping relationships, confidence and self-efficacy with the mentor role, attitudes toward children, expectations and motivation, and general well-being.

*Age*. The literature has pointed to some advantages of older mentors in long-term mentoring programs with or without a defined endpoint, although the findings are inconclusive. It should be noted that these studies varied in their sample size, reporters, and methodology, perhaps explaining these inconsistencies. Data on children from a 10-year longitudinal study revealed a small but significant positive correlation between mentor age and match length, indicating that older mentors had longer matches in U.S. mentoring programs [137]. Younger American mentors (aged 18–25) reported relatively more feelings of being overwhelmed due to role overload, exposure to risk-environment or opaque role boundaries, and feeling unappreciated and unsupported by the mentee’s parents [89]. American mentors who were newly married and in their late 20s tended to have shorter matches than did older mentors in BBBS programs [33]. However, a study evaluating a short-term American mentoring program did not find age to be a moderator [70]. Likewise, older mentors in BBBS America reported less self-efficacy as mentors or generated fewer relationship benefits as perceived by their mentees [136].

*Personal attributes*. Mentors’ confidence in the mentoring relationship and self-efficacy in the mentor’s role results in spending more time with the mentees [136], as well as closer and better relationships with them in short-term and long-term mentoring relationships with or without a precise ending point [136,138,139]. Mentors with more experience in helping roles with young people or working in allied professions (e.g., counselor, social worker, therapist) were found to be more effective than those with non-helping backgrounds, as reflected in meta-analyses with various types of programs [12,13]. These more experienced mentors reported higher relationship quality [140], mentoring self-efficacy [90], and experienced greater satisfaction within the mentoring relationship [141]. Mentors’ multicultural competence (i.e., awareness, beliefs, knowledge, and skills shaping the interactions with people from different ethnic minority groups) was a predictor of their satisfaction with the relationship and the program [142]. Conversely, mentors’ inability to bridge cultural differences appeared to be a pivotal contributor to the termination of matches [117].

Mentors in BBBS America who held mentees in positive regard and were capable of engaging authentically and empathically with them were more likely to facilitate the establishment of strong relationships [94,117]. In contrast, mentors holding negative attitudes toward children and adolescents often found it challenging to connect with or understand them [94,117]. Examining mentors from different mentoring programs with diverse duration, Gettings and Wilson [143] found that mentors’ commitment predicted relational maintenance strategies, such as positive communication and conflict management in mentoring. The perceived similarity between BBBS mentors’ ideal versus actual roles were significant predictors of mentors’ expressed intentions to preserve the relationship [144], whereas mentors’ feelings of frustration and ineffectiveness compared with their initial expectations were shown to account for early termination, as reported in qualitative studies [116,117,120].

A few, albeit contradictory, findings have related to intrapersonal factors, such as personality traits, well-being and relational history, of mentors who participated in short-term programs, thus calling for further exploration. For example, higher mentor conscientiousness, extraversion, and agreeableness were associated with closer mentor-mentee alliances reported by mentors [138]. Mentors with higher levels of depressive symptoms reported increased avoidance in the mentoring relationship and lower relationship satisfaction [145].

In contrast, mentors who reported having experienced higher levels of early life stress had mentees who reported greater satisfaction in the mentoring relationship and decreased relational anxiety [145]. These findings may indicate the need for a certain element of mentor vulnerability to exhibit empathy. Taken together, there is fertile ground for the further study of mentors’ personal attributes. 

Mentee characteristics. Mentors are not the sole actors in the mentoring interaction. Through reciprocal communication, mentees shape the mentoring relationship with their mentor. Mentee characteristics may be linked to the quality of the relationship or to the increased risk of premature termination, though much work remains to be done. To date, most studies have limited their attention to mentee background characteristics, such as age and risk status. 

*Age*. The ability and motivation of young people to forge close relationships with their mentors can vary as a function of their developmental status. Different developmental phases may accentuate various issues throughout the mentoring relationship. Nevertheless, most studies have addressed adolescence as a unitary stage, without differentiating it into phases (early, middle, or late). Findings from various American programs have indicated that mid-to late-elementary school-aged children and younger adolescents reported closer relationships with their mentors and tended to have more enduring matches than those characterizing older adolescents [33,53,113]. Early termination by mid- and late-adolescents is attributed mainly to adolescents striving for autonomy and independence. 

*Risk status.* Beyond age, mentees’ risk factors have been found to be associated with relationship dysfunction. For instance, family instability may increase the risk of early relationship termination by circumscribing the mentees’ ability to maintain continuous contact with their mentors, particularly in single-parent homes affected by higher than average rates of home moves or family environments characterized by intense conflict, drug use, and unsafe parenting [114]. Young people who have been referred for social services or have sustained emotional, sexual, or physical abuse are more likely to have premature closure rates in BBBS [33].

Children and adolescents with significant disruptions in their attachments to their primary caregivers (e.g., children of prisoners, children in foster care) may find it a challenge to engage in mentoring relationships, thus placing these relationships at higher risk than relationships with young people who do not fall into these populations [146]. Although two quantitative studies have found that mentored young people in foster care manifested improved social skills, mental health, quality of life, social skills, and greater trust in others than did foster care boys and girls in control groups [147,148,149] found that mentees in foster care had shorter matches than mentees not in foster care. Establishing strong mentoring relationships contributed to life skills development, as revealed in mentors’ and mentees’ interviews [108].

The presence of co-occurring risk factors may challenge the mentoring relationship’s sustainability and longevity, as suggested by some studies conducted in various mentoring programs with or without fixed ending points and duration across diverse programs. Risk factors in children have been thought to accumulate additively in a linear manner, such that low-risk exposure is associated with the most favorable outcomes, and high-risk exposure is associated with the worst outcomes. 

For example, in a study of 1310 children and adolescents, Herrera et al. [112] found that, although match quality, length, and frequency of meeting did not vary according to the level of risk, mentors who were paired with more risk-exposed mentees reported more challenges within the match. These challenges included frequent cancellations by the mentees, difficulty managing the mentees’ behavioral problems, and greater needs for program staff support. Similarly, secondary data analyses of an extensive database of 170 mentoring programs and 6468 matches from across the U.S. revealed that significant risk factors for premature match closure were delinquency, court involvement, and being gang-involved or at risk for gang involvement [113]. Family background, such as being in foster care, being an immigrant, and having an incarcerated parent, were also antecedents to premature closure. Furthermore, having academic problems, poor grades, and school attendance problems also increased the risk of premature closure [113]. Other factors significantly associated with premature terminations included risky health behaviors (e.g., substance use and adolescent pregnancy), as well as internalizing problems (e.g., anxiety, depression, and low self-esteem), and externalizing problems (e.g., behavior regulation difficulties and self-control problems [113,147].

Finally, several risk factors stemming from the mentees’ environment were reported to be negatively associated with the quality of the mentoring relationship in a sample of 455 Americans: economic adversity, family stress, and peer difficulties, but surprisingly, not individual risk, such as academic or behavioral problems, and mental health concerns [46]. Lengthier mentoring relationships were predicted by being from a low-income family (rather than from a very low-income family) among young people receiving outpatient mental health services [150]. However, behavioral difficulties predicted early termination in a study conducted in BBBS Canada [41].

Whereas the studies reviewed above point to a set of mentee risk circumstances as damaging to the mentoring process. However, DuBois et al.’s [13] meta-analysis reported a curvilinear relationship between risk and outcomes, suggesting that moderate risk exposure may be optimal for producing positive mentoring outcomes. They examined the effectiveness of mentoring among four groups of at-risk adolescents, based on high versus low levels of environmental risk (e.g., family conflict, poverty) or individual risk (e.g., behavioral, academic, social difficulties). Likewise, findings from a 12-week mentoring intervention for 90 American homeless adolescents showed that youth with a history of physical or sexual abuse attended more mentoring sessions [151].

*Relational history*. Feelings of suspicion, skepticism, resistance, and difficulty opening up, manifested throughout the relationships rooted in the youth’s painful history with their attachment figures, may be reenacted in the mentoring relationship [19]. Preliminary findings demonstrated that less maternal trust and quality communication predicted lower quality mentoring relationships among American adolescent female mentees in a short-term American program [152]. Early match terminations in BBBS Canada were less likely to occur when parents provided emotional support and when mentees’ parents or guardians perceived support from their network [41,111]. In contrast, securely attached mentees were more likely to forge close relationships with their mentors in a short-term Israeli [52] and a long-term American mentoring program without termination point [153] and exhibit greater improvement following the mentoring [52].

Male mentees who reported better relationships with their parents or guardians at baseline participated in matches that lasted at least one year in BBBS America [154]. American mentees with better preexisting adult relationships and stronger family ties and school bonds established higher-quality relationships with their mentors in a short-term mentoring program [54]. Finally, mentors’ support predicted positive changes in the mentees’ academic adjustment following their participation in BBBS Canada, primarily when the mentees had already reported considerable support from their mothers [155]. These findings should encourage researchers to question the assumption that mentoring constitutes a corrective experience for mentees (i.e., the compensatory model) and underscore the need to involve parents in the mentoring process.

*Personality*. There is a dearth of work examining the role of personality among mentees. However, findings from a short-term limited-time mentoring program in Israel suggest that mentees’ positive personality traits, such as extraversion, agreeableness, conscientiousness, and openness, were positively correlated with more positive expectations to emerge as a result of the mentoring. Agreeableness was also positively correlated with mentees’ perceived quality of the relationship in a short-term Israeli mentoring program [124]. Mentees’ external pressure to join the Canadian BBBS program and their reports of match difficulties were associated with a higher likelihood of early closure [41]. Future research should examine the bidirectional effects of mentors and mentees on the quality of the relationship and its dynamics, as documented in multiple reports and measures using dyadic analyses. 

*Mentoring Dyad.* Some of the factors shaping the mentoring relationship pertain to broader aspects of the mentoring dyad. These include culture, gender, program practices, and match characteristics.

*Culture*. Although culture can affect the goals and practices of the relationship [156], researchers have rarely examined whether the characteristics of the mentoring relationship are universal or culturally dependent [157,158]. Indeed, studies internationally have underscored the widespread notion of mentoring as a warm, caring relationship (see, for example, studies in Hong Kong—Chan and Ho, [64]: China—Chan et al. [58]; Glasgow—McArthur et al. [56,159]; Rwanda—[60]; UK—[107]; Sweden—[39]; and the Czech Republic—[160], with only passing attention paid to the role played by race and ethnicity in formal mentoring relationships [161,162,163,164,165]. Furthermore, questions about whether formal mentors from different cultures provide different kinds of support and how their mentees perceive this support have rarely been addressed [117,164,165]. To date, only few studies have directly addressed cultural differences. For instance, a comparison between 66 American mentoring programs and 50 European programs revealed that U.S. programs targeted high-risk populations and marginally focused on mitigation (i.e., prevention and coping with stressful situations), while European programs targeted immigrant and refugee populations and concentrated on inclusion (i.e., concentrating on promotion and positive youth development). The European programs considered mentoring a bidirectional tool for developing intercultural competence [157]. De Wit et al., [166] compared Aboriginal and non-Aboriginal mentees in BBBS Canada and reported that Aboriginals were significantly less likely than non-aboriginal adolescents to be in a long-term continuous mentoring relationship. However, Aboriginal mentees were significantly more likely than non-Aboriginal adolescents to report high-quality mentoring relationships characterized by feelings of closeness, warmth, trust, respect, and happiness, more regular weekly contact and monthly mentoring activities and exhibited better socioemotional functioning at the end of the intervention. The authors relate these findings to the fact that the Aboriginal culture emphasizes kinship and a search for meaning in social relationships.

Several qualitative studies on asylum seekers in Sweden and Australia have underscored the culturally specific needs of mentees to feel safe and protected, the need for social support and social capital, and their strong desire to fit in and move forward with their lives [167]. Garraway and Pistrang [107], stressed the need to combine dyadic and group settings of mentoring among African-Caribbean young people, as the African-Caribbean culture values collectivism and community relationships. Pryce et al. [163] examining a mentoring program in India, noted the importance of a symmetrical mentoring relationship to develop the mentee as a whole person and to see the mentee as reflecting the society’s collectivistic values. These preliminary findings suggest that adopting culturally informed theories and notions can contribute to a better understanding of the differences in goals, power dynamics, relationship quality, and outcomes across cultures [39,156,168,169]

*Race and ethnicity*. Theoretical writings [164] and qualitative studies [85,107,170] have suggested that race and ethnic similarity may comprise key facets of attraction and closeness, as shared culture is more likely to improve the strength and the length of the relationship by facilitating processes of idealization, role modeling, and identification. These positive outcomes are particularly manifest when non-judgment, advice-giving, and confidentiality are preserved [85] and when shared experiences of discrimination and shared socioeconomic status are integrated into the relationship [107]. Similarly, Raposa et al.’s. [17] meta-analysis indicated that same-race relationships lasted longer than cross-race matches, and cross-race relationships were more likely to end prematurely in BBBS America [33]. However, these proximal benefits did not translate into differences in terms of consistent outcomes [12,13,33,34,45,70,171]. With an eye to future research, the inclusion of culture or its inherent values as possible process-oriented variables may explain these inconsistencies.

*Gender*. The mentoring relationship is likely to be experienced and established differently by female and male mentors and mentees. For instance, gender-based theories pertaining to mentoring suggest that girls generally exhibit a more favorable response to mentoring because they place a greater emphasis on contact-focused goals [23,172]). However, other researchers have challenged this assumption, positing that both boys and girls have similar expectations [154].

Empirically, the quantitative findings concerning the influence of mentors’ and mentees’ gender are mixed. Note that, as BBBS interventions usually avoid making cross-sex matches, almost no data are available regarding the effect of cross-sex versus same-sex matches. 

*Mentors.* Early match terminations are less frequent for male than for female mentors in American programs [33,113,154]. In addition, male mentors reported stronger mentoring relationship quality [154]. Qualitative interviews conducted in BBBS America showed that female mentors were more likely to strive for a close relationship to develop quickly, whereas male mentors expected to engage mainly in fun activities [154].

*Mentees*. The findings on the duration of relationships for boys and girls are mixed. Whereas one study conducted in the context of BBBS America reported that girls’ relationships lasted significantly longer than those of boys [173], this finding was not replicated in BBBS Canada, where boys’ matches were more likely to last at least one year [111]. Furthermore, early terminations of the match were more likely among girls than among boys in BBBS America and Canada [33,41].

Inconsistent findings for the quality of the relationship have also emerged. Whereas girls in long-term relationships in BBBS America and in the short-term mentoring program were more satisfied with the relationship and rated it as more helpful than did boys [46,173], another study conducted on BBBS America found that boys, whose matches lasted at least one year, reported stronger mentoring relationships after three months than did girls whose matches lasted more than a year or less than a year [154].

Qualitative analyses conducted in BBBS America and Croatia and in a short-term mentoring program in the UK show that both male and female young people and male mentors had similar expectations for the relationship, mainly to engage in fun activities [154]. Interviews with adult male mentors and adolescent boys paired in BBBS America indicated that boys, similar to girls, valued emotional closeness in their mentoring relationships [117,154]. Same-gender pairing was reported to facilitate the relationship, mostly due to the greater likelihood of same-sex matches engaging in a wider spectrum of joint activities [160]. Whereas enduring male mentoring relationships were seen as potentially providing adolescent boys with models for positive masculinity, characterized by emotional disclosure and intimacy [107,119], quantitative analyses conducted in BBBS or in short-term mentoring programs in America did not find such benefits in same-gender matches when predicting match length [14] or the quality of the relationship [70]. Nevertheless, as noted, the vast majority of matches are same-sex, limiting the opportunity to compare dyads of different gender combinations; thus, the findings should be interpreted with caution.

*Match characteristics*. Although mentors’ and mentees’ similarity and perceived similarity in terms of fields of interest, temperament, personality traits, relational styles, and shared attitudes and values have been argued to be fundamental to the quality of the relationship and its outcomes, there is a striking dearth of research on similarity-based matching [14] This absence is surprising, given that the mentoring relationship is a dyadic phenomenon.

The lack of research notwithstanding, several studies have offered support to the critical role of match characteristics. For instance, in Eby et al.’s [174] meta-analysis, which summarized youth, academic, and workplace research on potential mentoring antecedents, mentees’ perceived similarity with their mentors in attitudes, values, beliefs, and personality was associated with overall mentoring satisfaction and instrumental and psychological support. When similarity in mentors’ and mentees’ interests was used as the primary matching criterion for programs, it served as a buffer against early termination in cross-race dyads [33]. De Wit et al. [37] found that mentees’ perceived similarity and identification with their mentors predicted the quality of the mentoring relationship in a study of 335 mentees in BBBS Canada. Contrary to expectation, Raposa et al. [14] found, in the context of BBBS America, that the lack of shared dislike of activities reported by both mentors and mentees predicted longer matches and fewer early terminations rather than mentors and mentees having either shared or conflicting interests in activities. These findings point to the need to further explore the role of shared characteristics of the dyad.

*Program Practices*. Program practices may influence the mentoring relationship incorporated within them [12,175]. In this respect, guidance and the program’s forms of outreach with the mentees’ parents and guardians have been identified as central practices that shape the relationship, as reported in DuBois et al.’s [12] meta-analysis. The reported impact of these practices is consistent with Keller’s systemic, ecological model of mentoring [176], which posits that the mentoring process is influenced by the external social networks that play a central role in scaffolding the development and the maintenance of the relationship. *Guidance*. Guidance is seen to be a key element accounting for the length and quality of the mentoring relationship [66]. Mentors in various types of American programs who received early match training were likely to meet their mentees more frequently, had a match that lasted at least 12 months, and had higher relationship quality ratings in mentee reports [112]. The amount and level of emotional and tangible regular assistance and perceived support offered by the program and mentors’ perceptions of the quality of their training, skill-building, support for efficacy, and mattering have been shown to positively correlate with mentors’ perceived quality of the mentoring relationship [46,141,177]), perceived meaning in volunteering [178], mentoring duration [112,179], and frequent meetings [112] in different American programs with diverse duration. The totality of benchmarks and standards implemented according to the Elements of Effective Practice for Mentoring, 3rd Edition, by the BBBS mentoring programs predicted the length of mentoring relationships [113,147]. A qualitative study of various mentoring programs reported that ongoing support and encouragement from program coordinators that facilitate flexible responses to mentee’s changing needs, provide the opportunity for reflection, and provide practical advice were found to be critical for repairing relationship conflicts and breakdowns [118]. Another qualitative study reported that early terminations were more likely to occur when mentors perceived the program caseworker in BBBS America involvement as either excessive or scanty [117].

*Ties with parents and guardians*. Parents’ and guardians’ support of mentoring can either facilitate or hinder the development and maintenance of the mentoring relationship [121]. Evidence gathered from short- and long-term mentoring programs suggests that for mentoring programs that specifically address parental involvement, the likelihood of early closure is lessened [41]. Similarly, parent/guardian satisfaction with the mentoring goals in BBBS America mentoring programs predicts match strength and duration, as derived from mentors’ and mentees’ reports, whereas parent/guardian dissatisfaction predicted match closure [180]. Mentor satisfaction with the mentee’s family regarding the emotional tone of the relationship, communication, cooperation, and appreciation predicted mentors’ satisfaction with the mentoring relationship, even after controlling for organizational support, mentors’ cultural competence, and other personal characteristics in a long-term American program [181]. Likewise, a high-quality parent-mentor relationship, as perceived by the mentors, predicted the quality of the mentee-mentor relationship in BBBS Canada [37].

Qualitative studies have provided insights into parents’ and guardians’ perceptions of their roles in the mentoring process. Parents described devoting substantial energies to cultivating their children’s mentor-mentee relationship, engaging in a range of roles, including mediator, coach, and collaborator [182,183,184]. However, both mentors and program staff reported judgment calls and considerable suspicions on the part of parents that could jeopardize the relationship [7,157,158,161]. These findings imply that further research is needed to explore how parent-mentor interaction can affect the mentoring relationship and can parents’ support of the mentoring relationship be enlisted.

## 4. Discussion

The current review illustrates the theoretical and empirical efforts undertaken over the last twenty years to capture the nature of CBM relationships. It demonstrates the importance of a single robust relational factor that includes the correlated dimensions of support, sensitivity, and trust to promote mentoring outcomes. Furthermore, the review highlights the impact of a balanced relationship in terms of the emotional tone, activities, structure, and hierarchy and underscores the need to adopt a more purposeful or intentional approach to shaping the goals of the relationship. Qualitative findings have yielded a triple typology, comprising the recreational, emotional, and catalyzing aspects of the relationship, which need further conceptualization and operationalization. The effect of duration on mentoring outcomes and mentoring quality emerged as significant, especially for long-term mentoring relationships and for mentoring programs with no definite ending point. 

Concerning the participants’ characteristics, the findings point to the benefits of mentors’ maturity in terms of age, attitudes toward underprivileged youth, experience, and of a certain measure of vulnerability when forming empathic relationships. Furthermore, some evidence indicated the advantage of the conditional assumption (i.e., the conditional assumption holding that mentees who have better relational history and have more positive personality traits exhibited higher relationship quality over the compensatory assumption). However, the review provides a certain optimism concerning the prospect of including mentees from various age groups and moderate risk status. Moreover, preliminary evidence on thriving and positive youth development as mediating variables may lead to a better understanding of the relationship’s course. Finally, the importance of training, parental involvement, and matching based on perceived similarities and similar interests emerged as important factors contributing to the quality of various types of mentoring relationships. 

## 5. Future Directions

The findings of the current review indicate a number of directions for further research. Specifically, considerable work is needed to expand and translate the field’s theoretical foundation and the qualitative findings into measurable concepts. For example, adopting concepts from the field of psychotherapy and parenting, such as the “common factor,” “transference and countertransference,” “self-disclosure” [106], and “parenting styles,” or practices could lead to the identification of additional mediators (e.g., regulation) and moderators (e.g., motivation), thus enabling a more comprehensive understanding of the mentoring interaction. In addition, mentoring theory and research could also benefit from conceptualizations borrowed from positive psychology, self-determination theory [185], prevention theory [186] and salutogenic theories. Conceptions, such as self-actualization, autonomy, purpose and meaning in life, optimism, hope, positive belief system, resilience, and a sense of coherence, can serve to pinpoint core mechanisms and typologies in mentoring relationships beyond the factor of closeness. Conceptualizations and assessments could also benefit from mapping different behaviors, activities, and strategies by eliciting the perspectives of experts, parents, mentees, mentors, practitioners, and former mentors and mentees.

Furthermore, the findings point to the need to probe the dynamics involved in mentoring and, in particular, the reciprocal influences between mentors and mentees, as well as processes of moderation and mediation. Moreover, to identify the mediating variables that shape the transactional nature of the mentor-mentee relationship more longitudinal data are needed from randomized control research designs that employ dyadic analyses, process models that take into account multiple individual and environmental characteristics in the same model [187]. Experimental research [40] is needed to examine whether the mediators that have been identified can be altered by manipulating the mentor, mentee, or context variables involved in mentoring. For example, future studies could assess whether changes in mentors’ sensitivity, attuned behaviors, or regulation (after training mentors to apply these behaviors) might modify mentees’ adjustment. Follow-up studies examining intra-individual [14] and interpersonal characteristics of mentee-mentor dyads, such as attachment security, feelings of helplessness, or self-worth, could facilitate examining whether and how these dyads are likely to evolve.

More fine-grained research is needed to shed light on the processes involved and the clear implications of the relationship at different developmental periods. For example, researchers could examine mentees across various developmental periods, as was done in natural mentoring [129], starting in middle childhood through the phases of adolescence (early, middle, and late), while examining different psychological needs, developmental tasks, and outcomes. Studies should also compare the influence of the core ingredients of the mentoring relationship across different cultural environments (e.g., collectivist vs. individualist) and populations (e.g., clinical, special needs) to identify the universal and culturally-specific components [156]. This kind of exploration could lead to a more in-depth understanding of how mentoring relationships are conducted and help shed light on the similarities and distinguishing features of various populations and contexts. Likewise, examining the moderating effect of gender by studying cross-sex and same-sex matches might help determine if and under which circumstances gender differences play a role in the mentoring relationship [188].

The use of multiple informants (parents, mentors, and mentees) and multi-methods in future research would help avoid problems associated with shared method variance. Specifically, it would be useful to examine the similarities and differences in informants’ perceptions and investigate their origins [26,187]. To disentangle what mentors actually say and do from how mentees perceive it, it is essential to use subjective measures of the mentoring relationship (diaries, projective measures, such as drawings and photographs, and interviews). By employing valid and detailed measures from various perspectives and researcher assessments, the gaps and inconsistencies manifest in previous research can be better resolved.

Using natural or laboratory-based observations of the dyadic interaction [189] can clarify the nature of mentors’ and mentees’ interactions, which may help detect the contribution of additional attributes of the relationship. These observations can be employed to assess relationship variables, such as acceptance versus rejection, closeness versus separateness, autonomy versus relatedness, hierarchy versus mutuality, attunement, and synchronization.

Finally, theoretically, the field of mentoring relationship can benefit from incorporating the notions of social justice, as well as critical and feminist thinking into mentoring relationships [23,150,190,191]. As mentors tend to belong to groups that occupy a privileged position in society, whereas mentees tend to belong to marginalized groups, and given the fact that mentoring relationships are inherently hierarchical, it is imperative for mentoring relationships to promote diversity, emphasize the standpoint of oppressed groups, and avoid recreating an oppressive power relationship and further marginalization [23,85,190]. The importance of these notions underscores the need to extend the conceptual prism governing the mentoring relationship beyond psychological dyadic thinking to encompass a broad sociological critical perspective in which power dynamics, ‘intersectionality,’ and critical reflection should be explored.

## 6. Practical Implications

Several pragmatic recommendations for mentoring practitioners emerged from the current review. Recruitment and selection of mentors and mentees is a significant task of mentoring organizations. The current review implies the need to assess mentors’ emotional maturity, cultural sensitivity, empathy, and attitudes toward at-risk youth and their families. In addition, training mentors toward shaping mentoring relationships characterized by warmth and trust, while adopting a balanced position integrating relational, recreational and goal-oriented approaches, was found to be a necessity. To achieve these, it is important to provide ongoing training to help mentors cope with relational difficulties resulting from mentees’ past relational histories and high-risk situation, as well as maintaining the relationship if motivation declines. Maintaining mentoring longevity for long-term mentoring relationships also evolved as an essential practice. Matching mentors and mentees according to similar interests and lack of significant dissimilarities, and treating mentoring rematches cautiously also surfaced as implications. Finally, in early-termination cases, the review pinpointed the importance of encouraging mentors and mentees to close the relationship properly rather than allowing the relationship to dissolve without formal closure. Nevertheless, the complexity of the findings and their nuances calls for caution in translating the results into applied guidelines.

## 7. Conclusions

Millions of children and adults are involved in CBM relationships worldwide. This review indicates that long enough, supportive, reliable, trustworthy, and balanced mentoring relationships in terms of goals, structure, and behaviors serve as building blocks in promoting mentees’ development and minimizing adversity. These aspects are conditioned by moderator factors, such as mentors’ and mentees’ characteristics, programs’ guidance, parental involvement, and match characteristics. The potential benefits of the mentoring relationship spotlights the need for more research in this field. More theoretical and methodical work is needed to broaden the conceptual perspective of the field and to identify the mediating processes and the measurement gaps within the theory and research. Despite these, the review provides support to the benefits of mentoring relationships providing practical implications of the reviewed work and suggestions for much needed future research.

## Figures and Tables

**Table 1:** Organization of the review.

Research Question	Topic	Sub-Topic	Sub-Topic
RQ1: Relationship Characteristics			
	Mentoring length and dosage		
	Quality of the relationship: Core ingredients		
	Types of activities		
	Mentoring Termination		
RQ2: Current Measurement Approaches			
RQ3: Mechanism through which the Mentoring Interaction Influences Relationship Quality and Duration	5Cs; PYD Parent-child relationship		
RQ4: Moderators Factors of Mentoring Relationships:	Mentor Characteristics	Age Intra-personal characteristics: Personality traits Well-being Relational history Personal attributes: Former experience Multicultural competence Attitudes toward children and adolescents Commitment	
	Mentee Characteristics	Age Risk Status Relational history Personality traits	
	Mentoring Dyads	Culture	Culture Race and Ethnicity
		Gender	Mentors Mentees
		Matching Criteria	
	Program Practices	Guidance	
		Ties with parents and guardians	

## Data Availability

Not applicable.

## Supplementary Materials

The following are available online at https://www.mdpi.com/article/10.3390/ijerph18115666/s1, Tables S1–S3.

## References

[1] Cavell T.A., Meehan B.T., Heffer R.W., Holladay J.J. (2002). The natural mentors of adolescent children of alcoholics (COAs): Implications for preventive practices. J. Prim. Prev..

[2] Rhodes J.E., Bogat G.A., Roffman J., Edelman P., Galasso L. (2002). Youth mentoring in perspective: Introduction to the special issue. Am. J. Community Psychol..

[3] DuBois D.L., Silverthorn N. (2005). Natural mentoring relationships and adolescent health: Evidence from a national study. Am. J. Public Health.

[4] Bruce M., Bridgeland J. (2014). The Mentoring Effect: Young People’s Perspectives on the Outcomes and Availability of Mentoring. A Report for Mentor: The National Mentoring Partnership.

[5] Hurd N.M., Zimmerman M.A. (2014). An analysis of natural mentoring relationship profiles and associations with mentees’ mental health: Considering links via support from important others. Am. J. Community Psychol..

[6] Van Dam L., Smit D., Wildschut B., Branje S.J.T., Rhodes J.E., Assink M., Stams G.J.J. (2018). Does natural mentoring matter? A multilevel meta-analysis on the association between natural mentoring and youth outcomes. Am. J. Community Psychol..

[7] Raposa E.B., Erickson L.D., Hagler M., Rhodes J.E. (2018). How economic disadvantage affects the availability and nature of mentoring relationships during the transition to adulthood. Am. J. Community Psychol..

[8] Schwartz S.E., Rhodes J.E. (2016). From treatment to empowerment: New approaches to youth mentoring. Am. J. Community Psychol..

[9] DuBois D.L., Karcher M.J., DuBois D.L., Karcher M.J. (2013). Youth mentoring: Progress and prospects for the 21st century. The Handbook of Youth Mentoring.

[10] Rhodes J.E., DuBois D.L. (2006). Understanding and facilitating the youth mentoring movement. Soc. Policy Rep..

[11] DuBois D.L., Keller T.E. (2017). Investigation of the integration of supports for youth thriving into a community-based mentoring program. Child Dev..

[12] DuBois D.L., Holloway B.E., Valentine J., Cooper H. (2002). Effectiveness of mentoring programs for youth: A meta-analytic review. Am. J. Community Psychol..

[13] DuBois D.L., Portillo N., Rhodes J.E., Silverthorn N., Valentine J.C. (2011). How effective are mentoring programs for youth?. A systematic assessment of the evidence. Psychol. Sci. Public Interest.

[14] Raposa E.B., Ben-Eliyahu A., Olsho L.E., Rhodes J. (2019). Birds of a feather: Is matching based on shared interests and characteristics associated with longer youth mentoring relationships?. J. Community Psychol..

[15] Jolliffe D., Farrington D.P. (2007). A Rapid Evidence Assessment of the Impact of Mentoring on Re-Offending: A Summary.

[16] Tolan P.H., Henry D.B., Schoeny M.S., Lovegrove P., Nichols E. (2014). Mentoring programs to affect delinquency and associated outcomes of youth at risk: A comprehensive meta-analytic review. J. Exp. Criminol..

[17] Raposa E.B., Rhodes J., Stams G.J.J., Card N., Burton S., Schwartz S., Hussain S. (2019). The effects of youth mentoring programs: A meta-analysis of outcome studies. J. Youth Adolesc..

[18] Rhodes J.E., Reddy R., Grossman J.B. (2005). The protective influence of mentoring on adolescents’ substance use: Direct and indirect pathways. Appl. Dev. Sci..

[19] Noam G.G., Malti T., Karcher M.J., DuBois D.L., Karcher M.J. (2013). Mentoring relationships in development perspective. Handbook of Youth Mentoring.

[20] Rhodes J.E., Spencer R., Keller T., Liang B., Noam G. (2006). A model for the influence of mentoring relationships on youth development. J. Community Psychol..

[21] Barrera M., Bonds D.B., DuBois D.L., Karcher M.J. (2005). Mentoring relationships and social support. The Handbook of Youth Mentoring.

[22] Stanton-Salazar R.D. (2011). A social capital framework for the study of institutional agents and their role in the empowerment of low-status students and youth. Youth Soc..

[23] Liang B., Spencer R., West J., Rappaport N. (2013). Expanding the reach of youth mentoring: Partnering with youth for personal growth and social change. J. Adolesc..

[24] Norton C.L., Watt T.T. (2014). Exploring the impact of a wilderness-based positive youth development program for urban youth. J. Exp. Educ..

[25] Lerner R.M., Napolitano C.M., Boyd M.J., Mueller M.K., Callina K.S., DuBois D.L., Karcher M.J. (2013). Mentoring and positive youth development. Handbook of Youth Mentoring.

[26] Deutsch N.L., Spencer R. (2009). Capturing the magic: Assessing the quality of youth mentoring relationships. New Dir. Youth Dev..

[27] Alfonso Y.N., Johnson S.L., Cheng T., Jones V., Ryan L., Fein J., Bishai D. (2019). A marginal cost analysis of a Big Brothers Big Sisters of America youth mentoring program: New evidence using statistical analysis. Child. Youth Serv. Rev..

[28] Stewart C., Openshaw L. (2014). Youth mentoring: What is it and what do we know?. J. Evid. Based Soc. Work.

[29] Schwartz S.E., Lowe S.R., Rhodes J.E. (2012). Mentoring relationships and adolescent self-esteem. Prev. Res..

[30] PRISMA PRISMA Procedure Transparent Reporting of Systematic Reviews and Meta-Analysis. http://www.prisma-statement.org/.

[31] Keller T.E., DuBois D.L., Karcher M.J. (2005). The stages and development of mentoring relationships. The Handbook of Youth Mentoring.

[32] Higley E., Walker S.C., Bishop A.S., Fritz C. (2016). Achieving high quality and long-lasting matches in youth mentoring programmes: A case study of 4Results mentoring. Child Fam. Soc. Work.

[33] Grossman J.B., Rhodes J.E. (2002). The test of time: Predictors and effect of duration in youth mentoring relationships. Am. J. Community Psychol..

[34] Park H., Yoon J., Crosby S.D. (2016). A pilot study of Big Brothers Big Sisters programs and youth development: An application of critical race theory. Child. Youth Serv. Rev..

[35] Erdem G., DuBois D.L., Larose S., De Wit D., Lipman E.L. (2016). Mentoring relationships, positive development, youth emotional and behavioral problems: Investigation of a mediational model. J. Community Psychol..

[36] Tierney J.P., Grossman J.B., Resch N.L. (2000). Making a Difference: An Impact Study of Big Brothers Big Sisters.

[37] De Wit D.J., DuBois D.L., Erdem G., Larose S., Lipman E.L. (2019). Predictors of mentoring relationship quality: Investigation from the perspectives of youth and parent participants in Big Brothers Big Sisters of Canada one-to-one mentoring programs. J. Community Psychol..

[38] Bodin M., Leifman H. (2011). A randomized effectiveness trial of an adult-to-youth mentoring program in Sweden. Addict. Res. Theory.

[39] Larsson M., Pettersson C., Skoog T., Eriksson C. (2016). Enabling relationship formation, development, and closure in a one-year female mentoring program at a non-governmental organization: A mixed-method study. Bmc Public Health.

[40] Ferro A., Wells S., Speechley K.N., Lipman E., De Wit D. (2014). The measurement properties of mentoring relationship quality scales for mentoring programs. Prev. Sci..

[41] De Wit D.J., DuBois D., Erdem G., Larose S., Lipman E.L., Spencer R. (2016). Mentoring relationship closures in Big Brothers Big Sisters community mentoring programs: Patterns and associated risk factors. Am. J. Community Psychol..

[42] Rhodes J.E., Schwartz S.E., Willis M.M., Wu M.B. (2017). Validating a mentoring relationship quality scale: Does match strength predict match length?. Youth Soc..

[43] La Valle C. (2015). The effectiveness of mentoring youth with externalizing and internalizing behavioral problems on youth outcomes and parenting atress: A Meta-analysis. Mentor. Tutoring Partnersh. Learn..

[44] Miller J.M., Barnes J.C., Miller H.V., McKinnon L. (2013). Exploring the link between mentoring program structure & success rates: Results from a national survey. Am. J. Crim. Justice.

[45] Gaddis S.M. (2012). What’s in a relationship? An examination of social capital, race and class in mentoring relationships. Soc. Forces.

[46] Weiler L.M., Boat A.A., Haddock S.A. (2019). Youth risk and mentoring relationship quality: The moderating effect of program experiences. Am. J. Community Psychol..

[47] Cavell T.A., Elledge L.C., Malcolm K.T., Faith M.A., Hughes J.N. (2009). Relationship quality and the mentoring of aggressive, high-risk children. J. Clin. Child Adolesc. Psychol..

[48] Cavell T.A., Hughes J.N. (2000). Secondary prevention as context for assessing change processes in aggressive children. J. Sch. Psychol..

[49] Sale E., Bellamy N., Springer J.F., Wang M.Q. (2008). Quality of provider-participant relationships and enhancement of adolescent social skills. J. Prim. Prev..

[50] Chapman C.M., Deane K.L., Harré N., Courtney M.G., Moore J. (2017). Engagement and mentor support as drivers of social development in the project K youth development program. J. Youth Adolesc..

[51] Goldner L., Mayseless O. (2009). The quality of mentoring relationships and mentoring success. J. Youth Adolesc..

[52] Goldner L., Scharf M. (2014). Attachment security, the quality of the mentoring relationship and protégés’ adjustment. J. Prim. Prev..

[53] Thomson N.R., Zand D.H. (2010). Mentees’ perceptions of their interpersonal relationships: The role of the mentor-youth bond. Youth Soc..

[54] Zand D.H., Thompson N., Cervantes R., Espritu R., Klagholz D., La Blanc L., Taylor A. (2009). The mentor-youth alliance: The role of mentoring relationships in promoting youth competence. J. Adolesc..

[55] Bowers E.P., Wang J., Tirrell J.M., Lerner R.M. (2016). A cross-lagged model of the development of mentor-mentee relationships and intentional self-regulation in adolescence. J. Community Psychol..

[56] Ng E.C.W., Lai M.K., Chan C.C. (2014). Effectiveness of mentorship program among underprivileged children in Hong Kong. Child. Youth Serv. Rev..

[57] Chesmore A.A., Weiler L.M., Taussig H.N. (2017). Mentoring relationship quality and maltreated children’s coping. Am. J. Community Psychol..

[58] Chan C.C., Guan Y., Choi P.Y. (2011). Chinese migrant children’s mental health and career efficacy: The roles of mentoring relationship quality and self-efficacy. Int. J. Disabil. Hum. Dev..

[59] Lau W.S., Zhou X.C., Lai S.M. (2017). The development of mentoring-relationship quality, future-planning style, and career goal setting among adolescents from a disadvantaged background. Psych J..

[60] Mukabutera A., Bizimana J.D.D., Owoeye O., Nzayirambaho M. (2013). Correlates of psychosocial outcomes among youth heads of households participating in mentoring programs: A study among Rwandan youths from Bugesera District. Vulnerable Child. Youth Stud..

[61] Silke C., Brady B., Boylan C., Dolan P. (2019). Relational dynamics in formal youth mentoring programmes: A longitudinal investigation into the association between relationship satisfaction and youth outcomes?. Child. Youth Serv. Rev..

[62] Pryce J.M., Gilkerson L., Barry J.E. (2018). The Mentoring FAN: A promising approach to enhancing attunement within the mentoring system. J. Soc. Serv. Res..

[63] Weiler L.M., Chesmore A.A., Pryce J., Haddock S.A., Rhodes T. (2019). Mentor response to youth academic support-seeking behavior: Does attunement matter?. Youth Soc..

[64] Chan C.C., Ho W.C. (2008). An ecological framework for evaluating relationship-functional aspects of youth mentoring. J. Appl. Soc. Psychol..

[65] Faith M.A., Fiala S.E., Cavell T.A., Hughes J.N. (2011). Mentoring highly aggressive children: Pre-post changes in mentors’ attitudes, personality, and attachment tendencies. J. Prim. Prev..

[66] Jarjoura G.R., DuBois D.L., Shlafer R.J., Haight K.A. (2013). Mentoring Children of Incarcerated Parents: A Synthesis of Research and Input from the Listening Session Held by the Office of Juvenile Justice and Delinquency Prevention and the White House Domestic Policy Council and Office of Public Engagement.

[67] Karcher M.J., Kuperminc G.P., Portwood S.G., Sipe C.L., Taylor A.S. (2006). Mentoring programs: A framework to inform program development, research, and evaluation. J. Community Psychol..

[68] Karcher M.J., Nakkula M.J. (2010). Youth mentoring with a balanced focus, shared purpose, and collaborative interactions. New Dir. Youth Dev..

[69] Karcher M.J., Herrera C., Hansen K. (2010). “I dunno, what do you wanna do?”: Testing a framework to guide mentor training and activity selection. New Dir. Youth Dev..

[70] Kern L., Harrison J.R., Custer B.E., Mehta P.D. (2019). Factors that enhance the quality of relationships between mentors and mentees during Check & Connect. Behav. Disord..

[71] Herrera C., Sipe C.L., McClanahan W.S. (2000). Mentoring School-Age Children: Relationship Development in Community-Based and School-Based Programs.

[72] Larose S., Savoie J., De Wit D.J., Lipman E.L., DuBois D.L. (2015). The role of relational, recreational, and tutoring activities in the perceptions of received support and quality of mentoring relationship during a community-based mentoring relationship. J. Community Psychol..

[73] Schwartz S.E., Rhodes J.E., Liang B., Sánchez B., Spencer R., Kremer S., Kanchewa S. (2014). Mentoring in the digital age: Social media use in adult-youth relationships. Child. Youth Serv. Rev..

[74] Christensen K.M., Hagler M.A., Stams G.J., Raposa E.B., Burton S., Rhodes J.E. (2020). Non-specific versus targeted approaches to youth mentoring: A follow-up meta-analysis. J. Youth Adolesc..

[75] Lee K.M., Krauss S., Suandi T., Hamzah A. (2016). Exploring the contribution of mentoring practices to mentee learning in a Malaysian youth development programme. Int. J. Adolesc. Youth.

[76] Keller T.E., DuBois D.L. (2019). Influence of program staff on quality of relationships in a community-based youth mentoring program. Ann. N. Y. Acad. Sci..

[77] Langhout R.D., Rhodes J.E., Osborne L.N. (2004). An exploratory study of youth mentoring in an urban context: Adolescents’ perceptions of relationship styles. J. Youth Adolesc..

[78] Weiler L.M., Zimmerman T.S., Haddock S., Krafchick J. (2014). Understanding the experience of mentor families in therapeutic youth mentoring. J. Community Psychol..

[79] Morrow K.V., Styles M.B. (1995). Building Relationships with Youth in Program Settings: A Study of Big Brothers Big Sisters.

[80] Donlan A.E., McDermott E.R., Zaff J.F. (2017). Building relationships between mentors and youth: Development of the TRICS model. Child. Youth Serv. Rev..

[81] Nakkula M.J., Harris J.T., DuBois D.L., Karcher M.J. (2013). Assessing mentoring relationships. Handbook of Youth Mentoring.

[82] Weybright E.H., Trauntvein N., Deen M.K. (2017). “It was like we were all equal”: Maximizing youth development using youth-adult partnerships. J. Park Recreat. Adm..

[83] Brady B., Dolan P., Canavan J. (2017). ‘He told me to calm down and all that’: A qualitative study of forms of social support in youth mentoring relationships. Child Fam. Soc. Work.

[84] Busse H., Campbell R., Kipping R. (2018). Developing a typology of mentoring programmes for young people attending secondary school in the United Kingdom using qualitative methods. Child. Youth Serv. Rev..

[85] Brinkman B.G., Marino S., Manning L. (2018). Relationships are the heart of the work: Mentoring relationships within gender-responsive programs for black girls. J. Fem. Fam. Ther..

[86] Brown K.D. (2017). Promoting Positive Social Development among African American Boys. https://search-proquest-com.ezproxy.haifa.ac.il/docview/1940351531?accountid=14544.

[87] Garcia-Molsosa M., Collet-Sabé J., Montserrat C. (2019). The role of mentoring in the schooling of children in residential care. Eur. J. Soc. Work.

[88] Hanham J., Tracey D. (2017). Evolution of mentoring relationships involving young male offenders transitioning from a juvenile justice center to the community. Youth Justice.

[89] Lakind D., Atkins M., Eddy J.M. (2015). Youth mentoring relationships in context: Mentor perceptions of youth, environment, and the mentor role. Child. Youth Serv. Rev..

[90] Lakind D., Eddy J.M., Zell A. (2014). Mentoring youth at high risk: The perspectives of professional mentors. Child Youth Care Forum.

[91] MacDonald K., Greggans A. (2010). ‘Cool friends’: An evaluation of a community befriending programme for young people with cystic fibrosis. J. Clin. Nurs..

[92] Radlick R.L., Mirkovic J., Przedpelska S., Brendmo E.H., Gammon D. (2020). Experiences and needs of multicultural youth and their mentors, and implications for digital mentoring platforms: Qualitative exploratory study. JMIR Form. Res..

[93] Smith C.A., Newman-Thomas C., Stormont M. (2015). Long-term mentors’ perceptions of building mentoring relationships with at-risk youth. Mentor. Tutoring: Partnersh. Learn..

[94] Spencer R. (2006). Understanding the mentoring process between adolescents and adults. Youth Soc..

[95] Spencer R., Liang B. (2009). “She gives me a break from the world”: Formal youth mentoring relationships between adolescent girls and adult women. J. Prim. Prev..

[96] Varga S.M., Deutsch N.L. (2016). Revealing both sides of the story: A comparative analysis of mentors and protégés relational perspectives. J. Prim. Prev..

[97] Weiler L.M., Zarich K.J., Haddock S.A., Krafchick J.L., Zimmerman T.S. (2014). A comprehensive model of mentor experiences: Perceptions, strategies, and outcomes. J. Community Psychol..

[98] Yanay-Ventura G., Amitay G. (2019). Volunteers’ practices in mentoring youth in distress: Volunteers as informal agents for youth. Child. Youth Serv. Rev..

[99] Vaclavik D., Sánchez B., Buehler K., Gray T., Rodriguez E. (2017). How to support me in connected learning: Youth perspectives on adult supportive behavior and its benefits. J. Community Psychol..

[100] Shelmerdine S., Louw J. (2008). Characteristics of mentoring relationships. J. Child Adolesc. Ment. Health.

[101] Spencer R., Pryce J., Barry J., Walsh J., Basualdo-Delmonico A. (2020). Deconstructing empathy: A qualitative examination of mentor perspective-taking and adaptability in youth mentoring relationships. Child. Youth Serv. Rev..

[102] Kazlauskaite V., Braughton J.E., Weiler L.M., Haddock S., Henry K.L., Lucas-Thompson R. (2020). Adolescents’ experiences of mentor alliance and sense of belonging in a site-based mentoring intervention. Child. Youth Serv. Rev..

[103] Dallos R., Carder-Gilbert H. (2019). Taking the stone from my heart: An exploration of the benefits of a mentoring programme (PROMISE) for children at risk of significant harm. Clin. Child Psychol. Psychiatry.

[104] Martin E., Bott C., Castellana L., Lancto K. (2017). Mrs. Doubtfire mentoring program: Helping children in residential care transition to bedtime. Resid. Treat. Child. Youth.

[105] Lester A.M., Goodloe C.L., Johnson H.E., Deutsch N.L. (2019). Understanding mutuality: Unpacking relational processes in youth mentoring relationships. J. Community Psychol..

[106] Dutton H. (2018). Mentor self-disclosure in youth mentoring relationships: A review of the literature about adults disclosing to non-familial adolescents in intervention settings. Adolesc. Res. Rev..

[107] Garraway H., Pistrang N. (2010). “Brother from another mother”: Mentoring for African-Caribbean adolescent boys. J. Adolesc..

[108] Osterling K.L., Hines A.M. (2006). Mentoring adolescent foster youth: Promoting resilience during developmental transitions. Child Fam. Soc. Work.

[109] Keller T.E., Pryce J.M. (2010). Mutual but unequal: Mentoring as a hybrid of familiar relationship roles. New Dir. Youth Dev..

[110] Zilberstein K., Spencer R. (2017). Breaking bad: An attachment perspective on youth mentoring relationship closures. Child Fam. Soc. Work.

[111] De Wit D.J., DuBois D., Erdem G., Larose S., Lipman E.L. (2016). The role of program-supported mentoring relationships in promoting youth mental health, behavioral and developmental outcomes. Prev. Sci..

[112] Herrera C., DuBois D.L., Grossman J.B. (2013). The Role of Risk: Mentoring Experiences and Outcomes for Youth with Varying Risk Profiles.

[113] Kupersmidt J.B., Stump K.N., Stelter R.L., Rhodes J.E. (2017). Predictors of premature match closure in youth mentoring relationships. Am. J. Community Psychol..

[114] Shlafer R.J., Poehlmann J., Coffino B., Hanneman A. (2009). Mentoring children with incarcerated parents: Implications for research, practice, and policy. Fam. Relat..

[115] Spencer R., Basualdo-Delmonico A. (2013). Termination and closure of mentoring relationships. Handbook of Youth Mentoring.

[116] Spencer R., Gowdy G., Drew A.L., McCormack M.J., Keller T.E. (2019). It takes a village to break up a match: A systemic analysis of formal youth mentoring relationship endings. Child & Youth Care Forum.

[117] Spencer R. (2007). “It’s not what I expected”—A qualitative study of youth mentoring relationship failure. J. Adolesc. Res..

[118] MacCallum J., Beltman S., Coffey A., Cooper T. (2017). Taking care of youth mentoring relationships: Red flags, repair, and respectful resolution. Mentor. Tutor. Partnersh. Learn..

[119] Spencer R. (2007). “I just feel safe with him”: Emotional closeness in male youth mentoring relationships. Psychol. Men Masc..

[120] Spencer R., Basualdo-Delmonico A., Walsh J., Drew A.L. (2017). Breaking up is hard to do: A qualitative interview study of how and why youth mentoring relationships end. Youth Soc..

[121] Spencer R., Keller T.E., Perry M., Drew A.L., Clark-Shim H., Horn J.P., McCormack M.J. (2019). How youth mentoring relationships end and why it matters: A mixed-methods, multi-informant study. Ann. N. Y. Acad. Sci..

[122] Bogat G.A., Liang B., Rigol-Dahn R.M. (2008). Stages of mentoring: An analysis of an intervention for pregnant and parenting adolescents. Child Adolesc. Soc. Work J..

[123] Pianta R.C., Steinberg M. (1992). Teacher-child relationships and the process of adjusting to school. New Dir. Child Adolesc. Dev..

[124] Goldner L. (2016). Proteges’ personality traits, expectations, the quality of the mentoring relationship and adjustment: A big five analysis. Child Youth Care Forum.

[125] Epstein S. (1983). The Mother-Father-Peer Scale.

[126] Armsden G.C., Greenberg M.T. (1987). The inventory of parent and peer attachment: Individual differences and their relationship to psychological well-being in adolescence. J. Youth Adolesc..

[127] Furman W., Buhrmester D. (1985). Children’s perceptions of the personal relationships in their social networks. Dev. Psychol..

[128] Shirk S.R., Saiz C.C. (1992). Clinical, empirical, and developmental perspectives on the therapeutic relationship in child psychotherapy. Dev. Psychopathol..

[129] Liang B., Spencer R., Brogan D., Corral M. (2008). Mentoring relationships from early adolescence through emerging adulthood: A qualitative analysis. J. Vocat. Behav..

[130] Spencer R., Rhodes J.E. (2005). A counseling and psychotherapy perspective on mentoring relationships. Handbook of Youth Mentoring.

[131] Rhodes J., Reddy R., Roffman J., Grossman J.B. (2005). Promoting successful youth mentoring relationships: A preliminary screening questionnaire. J. Prim. Prev..

[132] Liang B., Tracy A.J., Kenny M.E., Brogan D., Gatha R. (2010). The relational health indices for youth: An examination of reliability and validity aspects. Meas. Eval. Couns. Dev..

[133] Cuijpers P., Reijnders M., Huibers M.J. (2019). The role of common factors in psychotherapy outcomes. Annu. Rev. Clin. Psychol..

[134] Sulimani-Aidan Y., Melkman E., Hellman C.M. (2019). Nurturing the hope of youth in care: The contribution of mentoring. Am. J. Orthopsychiatry.

[135] Rhodes J.E., Grossman J.B., Resch N.L. (2000). Agents of change: Pathways through which mentoring relationships influence adolescents’ academic adjustment. Child Dev..

[136] Parra G.R., DuBois D.L., Neville H.A., Pugh-Lilly A.O., Povinelli N. (2002). Mentoring relationships for youth: Investigation of a process-oriented model. J. Community Psychol..

[137] Pedersen P.J., Woolum S., Gagne B., Coleman M. (2009). Beyond the norm: Extraordinary relationships in youth mentoring. Child. Youth Serv. Rev..

[138] Boat A.A., Weiler L.M., Bailey M., Haddock S., Henry K. (2019). Mentor’s self-efficacy trajectories during a mentoring program for at-risk adolescents. J. Prim. Prev..

[139] Ferro A., DeWit D., Wells S., Speechley K.N., Lipman E. (2013). An evaluation of the measurement properties of the Mentor Self-Efficacy Scale among participants in Big Brothers Big Sisters of Canada Community Mentoring Programs. Int. J. Evid. Based Coach. Mentor..

[140] Silverstein L.A. (2012). Are Good Intentions Enough? An Investigation of How Mentor Experiences and Expertise Affect Mentor-Mentee Relationship Development and Targeted Youth Outcomes. Ph.D. Dissertation.

[141] Martin S.M., Sifers S.K. (2012). An evaluation of factors leading to mentor satisfaction with the mentoring relationship. Child. Youth Serv. Rev..

[142] Suffrin R.L. (2014). The Role of Multicultural Competence, Privilege, Attributions, and Team Support in Predicting Positive Youth Mentor Outcomes. Coll. Sci. Health Theses Diss..

[143] Gettings P.E., Wilson S.R. (2014). Examining commitment and relational maintenance in formal youth mentoring relationships. J. Soc. Pers. Relatsh..

[144] Madia B.P., Lutz C.J. (2004). Perceived similarity, expectation-reality discrepancies, and mentors’ expressed intention to remain in Big Brothers/Big Sisters programs. J. Appl. Soc. Psychol..

[145] Preston E.G., Raposa E.B. (2019). A two-way street: Mentor stress and depression influence relational satisfaction and attachment in youth mentoring relationships. Am. J. Community Psychol..

[146] Spencer R., Basualdo-Delmonico A. (2014). Family involvement in the youth mentoring process: A focus group study with program staff. Child. Youth Serv. Rev..

[147] Stelter R.L., Kupersmidt J.B., Stump K.N. (2018). Supporting mentoring relationships of youth in foster care: Do program practices predict match length?. Am. J. Community Psychol..

[148] Taussig H.N., Culhane S.E. (2010). Impact of a mentoring and skills group program on mental health outcomes for maltreated children in foster care. Arch. Pediatrics Adolesc. Med..

[149] Taussig H.N., Weiler L.M., Garrido E.F., Rhodes T., Boat A., Fadell M. (2019). A positive youth development approach to improving mental health outcomes for maltreated children in foster care: Replication and extension of an RCT of the Fostering Healthy Futures Program. Am. J. Community Psychol..

[150] DuBois D.L., Herrera C., Higley E. (2018). Investigation of the reach and effectiveness of a mentoring program for youth receiving outpatient mental health services. Child. Youth Serv. Rev..

[151] Bartle-Haring S., Slesnick N., Collins J., Erdem G., Buettner C. (2012). The utility of mentoring homeless adolescents: A pilot study. Am. J. Drug Alcohol Abus..

[152] Williamson S., Lawrence E., Lyons M.D., Deutsch N.L. (2019). What mentees bring: Relationship characteristics pre-mentoring and mentoring relationship satisfaction. J. Early Adolesc..

[153] Zegers M.A., Schuengel C., van IJzendoorn M.H., Janssens J.M. (2006). Attachment representations of institutionalized adolescents and their professional caregivers: Predicting the development of therapeutic relationships. Am. J. Orthopsychiatry.

[154] Spencer R., Drew A.L., Walsh J., Kanchewa S.S. (2018). Girls (and boys) just want to have fun: A mixed-methods examination of the role of gender in youth mentoring relationship duration and quality. J. Prim. Prev..

[155] Larose S., Boisclair-Châteauvert G., De Wit D.J., DuBois D., Erdem G., Lipman E.L. (2018). How mentor support interacts with mother and teacher support in predicting youth academic adjustment: An investigation among youth exposed to Big Brothers Big Sisters of Canada programs. J. Prim. Prev..

[156] Goldner L., Scharf M., DuBois D.L., Karcher M.J. (2013). International and cross-cultural aspects in youth mentoring. The Handbook of Youth Mentoring.

[157] Preston J.M., Prieto-Flores Ò., Rhodes J.E. (2019). Mentoring in context: A comparative study of youth mentoring programs in the United States and continental Europe. Youth Soc..

[158] Zhou A.J., Lapointe É., Zhou S.S. (2019). Understanding mentoring relationships in China: Towards a Confucian model. Asia Pac. J. Manag..

[159] McArthur K., Wilson A., Hunter K. (2017). Mentor suitability and mentoring relationship quality: Lessons from the Glasgow Intergenerational Mentoring Network. J. Community Psychol..

[160] Komar M., Borić I.J. (2014). Gender aspects in mentoring children—The mentor’s perspective. Kriminol. Soc. Integr..

[161] Farruggia S.P., Bullen P., Solomon F., Collins E., Dunphy A. (2011). Examining the cultural context of youth mentoring: A systematic review. J. Prim. Prev..

[162] Kochan F. (2013). Analyzing the relationships between culture and mentoring. Mentor. Tutoring: Partnersh. Learn..

[163] Pryce J.M., Niederkorn A., Goins M., Reiland M. (2011). The development of a youth mentoring program in the south of India. Int. Soc. Work.

[164] Sanchez B., Colón Y. (2013). Race, ethnicity, and culture in mentoring relationships. Handbook of Youth Mentoring.

[165] Williams J.L., Deutsch N.L. (2016). Beyond between-group differences: Considering race, ethnicity, and culture in research on positive youth development programs. Appl. Dev. Sci..

[166] De Wit D.J., Wells S., Elton-Marshall T., George J. (2017). Mentoring relationships and the mental health of Aboriginal youth in Canada. J. Prim. Prev..

[167] Raithelhuber E. (2019). ‘If we want, they help us in any way’: How ‘unaccompanied refugee minors’ experience mentoring relationships. Eur. J. Soc. Work.

[168] Karcher M.J., Santos K.T. (2011). Promoting connectedness through developmental interventions: Adapting the Cross-Age Mentoring Program (CAMP) for youth in Asia. Asian J. Couns..

[169] Philip K. (2003). Youth mentoring: The American dream comes to the UK?. Br. J. Guid. Couns..

[170] Dawe A.M. (2017). What’s in It for Me? The Impact to Social Exchange Dynamics of Hispanic Males Serving as Mentors in Formal Youth Programs. Ph.D. Thesis.

[171] Rhodes J.E., Reddy R., Grossman J.B., Lee M.J. (2002). Volunteer mentoring relationships with minority youth: An analysis of same-versus cross-race matches. J. Appl. Soc. Psychol..

[172] Darling N., Bogat G.A., Cavell T.A., Murphy S.E., Sánchez B. (2006). Gender, ethnicity, development, and risk: Mentoring and the consideration of individual differences. J. Community Psychol..

[173] Rhodes J., Lowe S.R., Litchfield L., Walsh-Samp K. (2008). The role of gender in youth mentoring relationship formation and duration. J. Vocat. Behav..

[174] Eby L.T.D.T., Allen T.D., Hoffman B.J., Baranik L.E., Sauer J.B., Baldwin S., Evans S.C. (2013). An interdisciplinary meta-analysis of the potential antecedents, correlates, and consequences of protégé perceptions of mentoring. Psychol. Bull..

[175] Anastasia T.T., Skinner R.L., Mundhenk S.E. (2012). Youth mentoring: Program and mentor best practices. J. Fam. Consum. Sci..

[176] Keller T.E. (2005). A systemic model of the youth mentoring intervention. J. Prim. Prev..

[177] Marshall J.H., Davis M.C., Lawrence E.C., Peugh J.L., Toland M.D. (2016). Mentors’ perceived program support scale: Development and initial validation. J. Community Psychol..

[178] Keller T.E., Drew A., Clark-Shim H., Spencer R., Herrera C. (2020). It’s about time: Staff support contacts and mentor volunteer experiences. J. Youth Dev..

[179] Stump K.N., Kupersmidt J.B., Stelter R.L., Rhodes J.E. (2018). Mentoring program enhancements supporting effective mentoring of children of incarcerated parents. Am. J. Community Psychol..

[180] Shamblen S.R., Courser M.W., Schweinhart A.M., Thompson K. (2019). If momma ain’t happy with the mentoring relationship, ain’t nobody happy with the mentoring relationship: Parental satisfaction as a predictor of mentoring match strength and length. J. Community Psychol..

[181] Suffrin R.L., Todd N.R., Sánchez B. (2016). An ecological perspective of mentor satisfaction with their youth mentoring relationships. J. Community Psychol..

[182] Basualdo-Delmonico A.M., Spencer R. (2016). A parent’s place: Parents’, mentors’ and program staff members’ expectations for and experiences of parental involvement in community-based youth mentoring relationships. Child. Youth Serv. Rev..

[183] Keller T.E., Overton B., Pryce J.M., Barry J.E., Sutherland A., DuBois D.L. (2018). “I really wanted her to have a Big Sister”: Caregiver perspectives on mentoring for early adolescent girls. Child. Youth Serv. Rev..

[184] Spencer R., Basualdo-Delmonico A., Lewis T.O. (2011). Working to make it work: The role of parents in the youth mentoring process. J. Community Psychol..

[185] Madjar N., Cohen-Malayev M. (2013). Youth movements as educational settings promoting personal development: Comparing motivation and identity formation in formal and non-formal education contexts. Int. J. Educ. Res..

[186] Cavell T.A., Elledge L.C., DuBois D.L., Karcher M.J. (2013). Mentoring and prevention science. The Handbook of Youth Mentoring.

[187] DuBois D.L., Doolittle F., Yates B.T., Silverthorn N., Tebes J.K. (2006). Research methodology and youth mentoring. J. Community Psychol..

[188] Liang B., Bogat G.A., Duffy N., DuBois D.L., Karcher M.J. (2013). Gender in mentoring relationships. Handbook of Youth Mentoring.

[189] Pryce J., Deane K.L., Barry J.E., Keller T.E. (2020). Understanding youth mentoring relationships: Advancing the field with direct observational methods. Adolesc. Res. Rev..

[190] Albright J.N., Hurd N.M., Hussain S.B. (2017). Applying a social justice lens to youth mentoring: A review of the literature and recommendations for practice. Am. J. Community Psychol..

[191] Sánchez B., Pryce J., Silverthorn N., Deane K., Dubois D. (2018). Do mentor support for ethnic-racial identity and mentee cultural mistrust matter for girls of color? A preliminary investigation. Cult. Divers. Ethn. Minority Psychol..

