# Supplementary material for: Unpacking Community-Based Youth Mentoring Relationships: An Integrative Review
Source: Int J Environ Res Public Health. 2021 May 25;18(11):5666. doi: 10.3390/ijerph18115666 (PMC8198211; doi:10.3390/ijerph18115666)
Supplement: Supplementary file 1 [file ijerph-18-05666-s001.zip › Final Qualitative Table 22.10.20 after ed.pdf]

Table S2

Description of the Qualitative Studies

	Authors	Year	Aim	Country	Program	Duration Additional support	Population	Age	Sample	Design	Analytic approach	Main findings
1	Basualdo-Delmonico, & Spencer	2016	Examining expectations of parents' roles in the mentoring process by program staff, mentors, and parents	USA	BBBS	Long-term staff had an average of 3.6 years (3.99) of service	At-risk	Mentors 21-46 M = 28 (6.7) Parents 27-64 M=39 (8.2)	9 groups of program staff, mentors, and parents	In-depth interviews at two assessment points	Thematic Analysis	Mentors and staff were aligned in views and focused more on ways parents could disrupt the MR
2	Brady, Dolan, & Canavan	2017	Analyze the forms of social support in the MR	Ireland	BBBS	Short-term M = 5.2 months at 1st interview, M = 12.7 months at second interview	At-risk	Mentees M = 12 at time of referral Mentors M = 33 at recruitment	40 10 mentees, 10 parents, 10 mentors and 10 caseworkers	Semi-structured interviews at two assessment points	Thematic Analysis	Social support includes emotional support, esteem support, advice support and companionship
3	Brinkman, Marino, & Manning	2018	Explore the MR between adult staff and black girls	USA	Gender-responsive agency	Not specified	Black at-risk girls	Mentees 13-18 Mentors 24-59	10 dyads of adolescents and their mentors	Semi-structured interviews	Grounded Theory	Positive relationships characterized by integrity and trustworthiness, perceived support, and role-modeling
4	Brown	2017	Explore aspects of the MR	USA	Boys to Men	Diverse A few months to a few years	At-risk boys	11-17	14 mentors 4 mentees	Observa-tional data Focus groups Interviews	Thematic Analysis	Mentees' success was positively impacted by ability to trust mentors and the belief that mentors had an understanding of their lives.
5	Busse, Campbell, & Kipping	2018	Identify typology of mentoring	UK	23 mentoring organizations	Diverse A few months – several years	Secondary school adolescents	11-16	23 program managers	Telephone interviews	Grounded Theory	Personal/ developmental versus academic and employability

	Authors	Year	Aim	Country	Program	Duration Additional support	Population	Age	Sample	Design	Analytic approach	Main findings
												mentoring programs
6	Donlan, McDermott, & Zaff	2017	Explore aspects of MR development	USA	Youth Development Program (YDP)	Long-term (several years)	Low income, predominantly African American teens	Mentors and staff 27-63 Mentees 11-18	31 mentors 13 program staff 27 mentees	Focus groups	Grounded Theory	Five key features shape the relationship: The Right Who, Respect, Information gathering, Consistency, and Support
7	Dawe	2017	Explore aspects of MR in Hispanic populations	USA	Coachella Valley Youth Leadership and BBBS	Long-term Varied	Hispanic mentoring males	18-60	14 mentors	In-depth interviews	Inductive Theory	Hispanic male mentors set high expectations for themselves as mentors. Mentoring provides personal and professional development benefits.
8	Garcia-Molsosa, Collet-Sabé, & Montserrat	2019	Define the role and the contribution of the mentor to mentees' outcome, detecting the differences between mentors and other supportive adults	Austria, Croatia, France, Germany Spain	Erasmus+ K102 European social mentoring project	Not specified	Children/teens in residential care	Mentees 12-17 Mentors M = 34.2 range: 25-74 years	75 mentees 75 caregivers 69 teachers 62 mentors	Focus groups Reports analysis	Thematic Analysis	Providing supportive, caring, trustful and steady relationships, mentoring may create opportunities for the well-being and resilience of children/teens in residential care
9	Garraway & Pistrang	2010	Exploring the role of mentoring in promoting African-Caribbean adolescents' engagement in mental health services	UK (London)	A community-based mentoring scheme set up in partnership with a Child and Adolescent Mental Health Service (CAMHS)	Diverse between 5 months and 3 years	African-Caribbean adolescent boys with psychological and behavioral difficulties	12-17	13 mentees 5 mentors	Focus groups and Interviews	Interpretative Phenomenological Analysis	Strong, emotional bonds were formed between boys and mentors, facilitated by the perception of shared life experiences; boys were able

	Authors	Year	Aim	Country	Program	Duration Additional support	Population	Age	Sample	Design	Analytic approach	Main findings
												to show their vulnerabilities and accept support.
10	Hanham & Tracey	2017	Understanding the nature of the transition from a juvenile justice center to the community and the psychosocial and instrumental aspects of mentoring	Australia	Program accompanying the transition from a juvenile justice center to the community	Long-term (min 1 year)	Male teens in transition from a juvenile justice center to the community	16-19	13 mentees	Interviews over three waves	Thematic Analysis	Pre-release mentees valued the mentor as a guide, confidant and "watchdog". Post-release needed mentors to be reliable and help with occupational and educational opportunities.
11	Keller et al., 2018	2018	Assessing caregivers' perspectives and priorities	USA	Half in BBBS, half in BBBS GirlPOWER!	Long-term (1 year at interview) GirlPOWER! Also involves group-based, health-focused and gender-specific meetings	Early adolescent girls	M = 11.90 10-14	20 caregivers	Semi-structured interviews	Grounded Theory	Caregivers expected mentors to support mentees as confidants and conduits to opportunities and services. They noted mentoring reinforced their parenting.
12	Komar & Boric	2014	Exploring the role of gender to the MRQ	Croatia	Mentors in the Community, implementation of BBBS in Croatia	Short-term (min 3 months)	At-risk	19-30	20 mentors	Focus groups of mentors	Thematic Analysis	Girls met with mentors more frequently. Boys rated MR as more successful and stable than girls. Mentors indicated that gender interacted with age and mentee's personality when affecting the MR.

	Authors	Year	Aim	Country	Program	Duration Additional support	Population	Age	Sample	Design	Analytic approach	Main findings
13	Lakind, Atkins, & Eddy	2015	Exploring how mentors' perceptions of their mentees and mentee environments shape the mentoring role	USA	Friends of the Children (FOTC)	Long-term M = 3.8 years (1.7) 1.7-7.7 years	At-risk	M = 33.1 25-49	9 mentors	In-depth interviews	Grounded Theory	Mentors perceived the mentees' problems as byproducts of adverse environments, and some saw themselves as an antidote to it. They described role overload and blurred role boundaries.
14	Lakind, Eddy &, Zell	2014	Examine the role perceptions of professional mentors serving at-risk youth	USA	Friends of the Children (FOTC)	Long-term M = 3.8 years 1.7-7.7 years	At-risk	M = 33.1 25-49	9 mentors	Semi-structured	Thematic Analysis	Mentors reported professionalism enabled them to build expertise, provided them with credibility and authority, and contributed to their long-term commitment and ability to develop deep relationships with mentees.
15	Larsson, Pettersson, Skoog, & Eriksson	2016	Understanding mentees' experiences of the relationship process	Sweden	The Girls' Zone	Diverse	At-risk girls	M = 18.1 (2.8) 15-26	52 mentees	Mixed-method	Descriptive statistics Content Analysis	MRs started with nervousness and ambivalence, then developed authentic, non-hierarchical MRs. Closure accompanied by feelings of abandonment and strength.
16	Lester, Goodloe, Johnson, & Deutsch	2019	Unpacking relational processes in youth	USA	The Young Women Leaders Program	Short-term (school year) Group meeting for	At-risk girls	Mentees: middle school Mentors: 20-30	43 dyads of mentors and mentees	In-depth interviews	Thematic Analysis	Mutuality in MR is defined as a combination of shared

	Authors	Year	Aim	Country	Program	Duration Additional support	Population	Age	Sample	Design	Analytic approach	Main findings
			mentoring relationships			skill-building						relational excitement (desire to invest) and experiential empathy (mentors sharing their own experiences in order to connect).
17	MacCallum Beltmanb, Coffeyc, & Cooper	2017	Identifying early warning signals of early termination	Australia	4 programs (2 CBM, 2 SBM)	Diverse A few months – several years	At-risk	12-18	6 mentors 6 coordinators 4 mentees	Semi-structured interviews	Thematic Analysis	Red flags identified were mismatched expectations, lack of connection, lack of support from family, mentors ignoring program guidelines and cancelling at short notice. Restoring MR was possible using the provision of support for mentors to make changes in MR.
18	MacDonald & Greggans	2010	Evaluating the impact of CBM program on a group of mentees with a chronic illness	UK (Scotland)	The Butterfly Trusts (CBM): community youth mentoring youth with chronic illness	Diverse 1 month - 2 years	Young people with cystic fibrosis	8-18	10 mentees/ parents 3 mentors 4 key people	Semi-structured interviews, Focus groups	Thematic Analysis	MR offered a distraction from illness, respite for careers and a helpful environment to raise personal issues.
19	Martin, Bott, Castellana, & Lancto	2017	Explore mentoring contribution and experience	USA	Mrs. Doubtfire's mentoring program	Short-term (6 weeks)	Children in residential care	Mentees 6-11 Mentors M = 55 Staff 20-60	7 mentees 15 mentors 12 staff members	Survey Interviews and focus groups	Thematic Analysis	Mentors reported greater awareness and sensitivity to challenges children face.

	Authors	Year	Aim	Country	Program	Duration Additional support	Population	Age	Sample	Design	Analytic approach	Main findings
												They felt proud and committed. Children showed improvement in behavior, self-esteem and relationships with adults.
20	McArthur, Wilson, & Hunter	2016	Describing the relational suitability of mentors	UK (Scotland)	The Glasgow Intergenerational Mentoring Net-work	Short-term (school year)	At-risk	Not provided	3 dyads of mentors and mentees	In-depth interviews	Case examples	Providing mentors with training and support contributed to successful MR. Training should focus on relational skills and understanding children's social and cultural context.
21	Osterling& Hines	2006	Describing mentoring experience		The Advocates to Successful Transition to Independence' program	Long-term Two-years	Foster-care	Mentors: 47.1 Mentees: 16.3	18 mentors 52 mentees	Qualitative survey, interviews and focus groups		Mentoring relationship which concentrates mainly on advocacy can serve as a strategy for skill-building and as a resource needed for successful transition out of foster care and into adulthood
22	Pryce, Gilkerson, & Barry	2018	Exploring the effectiveness of the Mentoring FAN (Facilitating Attuned Interactions) in enhancing	USA	2 mentoring programs providing a wide range of mentoring styles, including SBM and CBM, e-mentoring, and	Long-term M = 5.8 years of experience in mentoring	Not specified	Staff M = 40 Mentors not provided	28 program staff 27 mentors	Mixed method Longitu-dinal pre/post question-naire	Open Coding using Nvivo	Mentoring staff experienced increases in empathy, attunement, listening ability and collaboration in

	Authors	Year	Aim	Country	Program	Duration Additional support	Population	Age	Sample	Design	Analytic approach	Main findings
			interpersonal attunement among mentoring staff		workplace mentoring					In-depth interviews		the supervisory relationship.
23	Pryce, Niederkorn, Goins, & Reiland	2011	Develop a culturally - informed manual in India	India (Tamil Nadu)	Youth development program	Not specified	At-risk	11-18	26 mentees<br>10 mentors<br>14 program alumni<br>3 program staff	Mixed method<br>Focus groups<br>Retro-spective question-naire and interviews	Content analysis	Mentors were shared by mentees and all parties expressed expectations for MR to be communal. Mentors viewed their role as helping mentees develop as a whole person. It was important to include members of the community in the development and implementation of the program.
24	Raithelhuber	2019	Describe how asylum seeking mentees perceive their relationships and make sense of mentoring	Austria	Godparenthoods for URMs	Not specified	Seeking asylum mentees	14-18	18 mentees	Group interviews	Content Analysis	Mentees described the MR as providing social support and social capital: improving communication skills and granting access to institutions and resources.
25	Shelmerdine, & Louw	2008	Provide in-depth qualitative information about relationship processes	South Africa	BBBS	Long-term 1 year	At-risk	Mentees 9-16<br>Mentors 20’s – 40’s	8 dyads of mentors and mentees	Semi-structured interviews	Content Analysis	Program was able to give rise to friendships for their own sake that could break negative cultural constructions viewing

	Authors	Year	Aim	Country	Program	Duration Additional support	Population	Age	Sample	Design	Analytic approach	Main findings
												mentors as having abilities mentees lack or a mission to change mentees' behavior
26	Smith, Newman-Thomas, & Stormont	2015	Understand mentors' perspective of MR processes that build resiliency and form a positive MR	USA	Diverse: CBM and SBM	Long-term 10-40 years' experience in mentoring	At-risk	Not specified	12 mentors	Focus group	Content Analysis	Mentees need supportive acceptance and consistency to form a healthy positive MR. Mentors believed they can enhance mentees' resilience by teaching and modeling social literacy and future visions.
27	Spencer	2006	Provide information about MR processes	USA	BBBS	Diverse	At-risk	Mentees 12-17 Mentors 25-55	24 dyads	Semi-structured interviews	Interpretive Analysis	Forming a deep MR depended on joint commitment and emotional involvement that included empathy, authenticity and engagement.
28	Spencer	2007	Understand relationship failure	USA	BBBS	Short-term 1-11 months	At-risk	10-13 19-47	11 mentees 20 mentors	Semi-structured interviews	Inductive and Constructive Analysis	Six factors contributed to early termination: mentor abandonment, perceived lack of mentee's motivation, unfulfilled expectations, deficiencies in mentor's relational skills, family

	Authors	Year	Aim	Country	Program	Duration Additional support	Population	Age	Sample	Design	Analytic approach	Main findings
												interference and inadequate agency support.
29	Spencer	2007	Provides an in-depth examination of close enduring MR between male adolescents and adults	USA	BBBS	Long-term 1-6 years M = 2.88 (1.59)	At-risk boys	Mentees 12-16 M = 13.83 (1.64) Mentors 25-48 M = 35.58 (5.92)	12 dyads	Semi-structured interviews	Thematic Analysis	Mentors wanted to be emotionally connected and role models. MR provided a safe space for emotional vulnerability and support and helped mentees manage anger more effectively.
30	Spencer & Basualdo-Delmonico	2014	Provide in-depth understanding of family involvement and its influence on MR	USA	BBBS	Long-term M = 5 years (3.85)	At-risk	Mentees served by programs 6-18 Staff M = 40 (11.63)	39 Staff members from 24 agency affiliates	Focus groups	Thematic Analysis using Atlas	Three approaches involving families in the mentoring process emerged: involving, engaging and serving, and collaborating.
31	Spencer, Basualdo-Delmonico, Walsh, & Drew	2017	Study MR endings	USA	BBBS	Diverse 2-24 months	At-risk	Mentees 9-15 M=12.5 (1.3) Mentors 20-55 M=27 (7.01) Parents 29-61 M=39.4 (6.9)	42 mentees 38 mentors 42 parents/ guardians	Semi-structured interviews	Thematic Analysis	Reasons for termination were changes in life circumstances, mentee dissatisfaction, mentor dissatisfaction, gradual dissolution and mentor abandonment.
32	Spencer, Basualdo-Delmonico, & Lewis	2011	Provide in-depth understanding of parents' involvement	USA	BBBS	Short-term Less than 1 year	At-risk	30-52 M = 40 (7.05) Mentees 9-14	13 parents	Semi-structured interviews	Thematic Analysis	Parents had hopes and expectations for the MR, trust and satisfaction with the mentor

	Authors	Year	Aim	Country	Program	Duration Additional support	Population	Age	Sample	Design	Analytic approach	Main findings
								M = 11.58 (1.51)				was important to them, roles of parents as collaborators or mediators for MR and cultural differences between mentors and mentees were also discussed.
33	Spencer, Gowdy, Drew, McCormack, & Keller	2019	Study premature MR endings	USA	BBBS	Diverse 1-18 months	At-risk	Mentees M = 11.6 (1.8) Parents M = 41.6 (10.4) Mentors M = 32.4 (9.1) Staff M = 32.2 (9.1)	36 mentees 36 Mentors 36 parents/ guardians 22 staff members	Semi-structured interviews	Thematic Analysis	A strong MR is necessary but not sufficient to withstand disruptions in other relationships in the system. Agency contextual factors play a critical role in sustaining MR.
34	Spencer, & Liang	2009	Study MR process in same- sex female matches	USA	BBBS	Long-term 2.5-11 years M = 5.08 (2.68)	At-risk	Mentees M=14.83 (1.59) Mentors M=38.67 (10.30)	12 mentees 12 mentors	Semi-structured interviews	Thematic Analysis	MR described as providing engaged and authentic emotional support, an opportunity to develop new skills and confidence and companionship that provided stress relief.
35	Vaclavik, Sánchez, Buehler, Gray, & Rodriguez	2017	Explore youth-staff relationships	USA	Five community-based, OST (out-of-school) programs	Not specified	At-risk	15-21 M = 17.35 (1.72)	26 mentees	Semi-structured interviews in focus groups	Grounded Theory	Mutual respect, genuine interest, ongoing communication and friendship enabled MR development. Mentees described MR

	Authors	Year	Aim	Country	Program	Duration Additional support	Population	Age	Sample	Design	Analytic approach	Main findings
												as providing guidance, connection and support. MR outcomes were social capital, empowerment and a sense of acceptance and validation.
36	Varga & deutsch	2016	Examine characteristics linked to mentoring satisfaction	USA	YWLP (Young Women Leaders) Program	Short-term (school year) Group meetings	At-risk girls	Mentees M=13 Mentors M=20.5	72 couples of mentors and mentees	Semi-structured interviews in focus groups as well as completing MRQ questionnaire.	Thematic Analysis	Authenticity, companionship, collaboration, and identification were associated with meaningful MR. Misattunement, was the most powerful distinguisher between high and low satisfaction dyads
37	Weiler, Zarich, Haddock, Krafchick, & Zimmerman	2014	Explore mentors' experiences	USA	Campus Corp	Short-term 12 weeks	At-risk	Mentees 11-18 Mentors 18-50 M = 21.07 (3.11)	123 mentors	Focus groups, weekly reflection journals	Grounded Theory	A focus on role, positive attitudes, communication and reflection provided personal growth, improved skills and professional development.
38	Weiler, Zimmerman, Haddock, & Krafchick	2014	Explore the experience of Mentor Families - (three to four pairs of mentors and mentees meeting together)	USA	Campus Corps program	Short-term 12 weeks	Adolescents at risk for delinquency	Mentees 10-18 M = 15.21 (1.93) Mentors 18-50 M = 21.30 (4.26)	212 mentors 87 mentees	Interviews Focus-groups	Grounded Theory	Mentor families provide support and supervision for mentors, a sense of belongings for both mentors and mentees and an opportunity for

	Authors	Year	Aim	Country	Program	Duration Additional support	Population	Age	Sample	Design	Analytic approach	Main findings
												mentees to grow and learn.
38	Weybright, Trauntvein, & Deen		Explore mentees' experiences	USA	4-H Youth Development program	Short-term 9 months	At-risk	14-18	29 mentees	Group and individual interviews and activity observation	Thematic Analysis	Mutual respect, autonomy teaching, adult coaching, shared responsibilities, friendship and having fun contributed to positive MR.
39	Yanay-Ventura, & Amitay	2019	Explore volunteer mentors' strategies	Israel	13 programs: boarding schools, youth villages, hostels and NGOs for youth in distress	Long-term 1-10 years volun-teering as mentors	Maltreated, in distress in youth villages	25-40	28 mentors (2/3 of whom defined themselves as former youth in distress)	Semi-structured interviews	Thematic Analysis	Strategies described: honesty and directness, listening, informal activities, refraining from judgment and containing anger, bridging between youth and caregivers, and cultivating a realistic sense of self-efficacy as volunteers.

Note MR=mentoring relationship, RQ= relationship quality, MRQ=mentoring relationship quality. Short-term<12 months, Long-term ≥12 months
