# Supplementary material for: Unpacking Community-Based Youth Mentoring Relationships: An Integrative Review
Source: Int J Environ Res Public Health. 2021 May 25;18(11):5666. doi: 10.3390/ijerph18115666 (PMC8198211; doi:10.3390/ijerph18115666)
Supplement: Supplementary file 1 [file ijerph-18-05666-s001.zip › final quantitative Table 6.2.21.pdf]

Table S1

Description of the Quantitative Studies

	Authors	Year	Aim	Country	Program	Duration Additional support	Population	Age (M/range)	Sample size	Design	Reporters	Main findings
1	Bodin & Leifman	2011	Study the effects of the program targeting substance use, delinquency, emotional and behavioral problems, social competence, quality of life, and school performance.	Sweden	Mentor Foundation	Long-term 12 months	Low-risk	14	128 treatment 65 control 63	RCT Longitudinal Baseline/post	Children Parents	Youth who met with their mentor for a full program period were significantly more likely than non-completers to report liking, trusting, and getting help from their mentors.
2	Boat, Weiler, Bailey, Haddock, & Henry	2019	Examine how changes in mentor self-efficacy are associated with mentor alliance, examine how mentor personality traits and mentee risk are related to changes in mentor self-efficacy.	USA	Campus Connection	Short-term 12-weeks	At-risk	M=14.1 (1.76) 11-18	238	Quai-experimental Longitudinal Baseline/post	Children Mentors	Mentors from the increased self-efficacy group exhibited greater mentoring alliance relative to the decreased group.
3	Bowers, Wang, Tirrell, & Lerner	2016	Describe the bidirectional relations between mentoring relationships (length and closeness) and youth self-regulation	USA	15 youth-serving programs	Not available	At-risk	M= 14.14 (2.25) 10-21	415 mentees	Quasi-experimental Longitudinal Baseline/post	Children Mentors	MRQ predicted growth in the mentees' self-regulation

	Authors	Year	Aim	Country	Program	Duration Additional support	Population	Age (M/range)	Sample size	Design	Reporters	Main findings
4	Cavell, Elledge, Malcolm, Faith, & Hughes	2009	Examine the extent to which relationship quality predicts outcomes for aggressive children in CBM and SBM mentoring programs	USA	PrimeTime (CBM) versus Lunch Buddy (SBM)	Long-term two academic years. Child skills training and consultation for parents and teachers	Aggressive elementary school students	CBM: M= 8.19 (.62) SBM: M=8.12 (.68)	145 mentees 70 CBM 75 SBM	Random clinical study Longitudinal Baseline/post	Children Mentors Parents Teachers	MRQ interacted with treatment in predicting changes in parent-rated externalizing behavior for CBM children.
5	Cavell & Hughes	2000	Examine the extent to which RQ and social cognition predict outcomes for aggressive children in two programs	USA	PrimeTime versus standard CBM	Long-term 16 months. Child skills training and consultation for parents and teachers	Aggressive elementary school students	PrimeTime: children M= 7.55 (.84) Standard CBM: M=7.54 (.74)	62 31 Prime Time 31 CBM	Random clinical study Longitudinal Baseline/post and follow up	Children Mentors Parents Teachers Peers	PrimeTime children's perceptions of their MRQ predicted parent-rated aggression at post-treatment. Mentors' ratings of the RQ predicted teacher-rated aggression at follow-up.
6	Chan, Guan, & Choi	2011	Examine whether relationship quality predicts mental health and career efficacy above and beyond relationship quality with parents	China	Mentorship program in Beijing	Not specified	Migrant children	M= 15.00 (1.72)	114	Cross-sectional	Children	MRQ predicts career efficacy and mental health.
7	Chan & Ho	2008	Identify factors that promote the mentoring contribution using an ecological approach	Hong-Kong	The ICM preventive intergenerational program	Long-term 18 months	At-risk	M=13.5 (1.4) 11-17	48	Mixed methods	Children Mentors Staff members	Relationship asymmetry predicted lower levels of perceived mentoring contribution.
8	Chapman, Deane, Harré, Courtney, & Moore	2017	Identify program components that promote social gains	USA	Project K	Long-term 14 months Outdoor adventure experiences	Low academic and social self-efficacy	13-15	327	Quasi-experimental Longitudinal, baseline/post	Children	Mentoring support predicted mentees' increased social self-efficacy and

	Authors	Year	Aim	Country	Program	Duration Additional support	Population	Age (M/range)	Sample size	Design	Reporters	Main findings
						participating in community activities						sense of community.
9	Chesmore, Weiler, & Taussig	2017	Identify program components that promote coping skills	USA		Short-term 9 months Skilled-based training	Maltreated children placed in foster care	M=10.3 (.92) 11-13	154	Quasi-experimental Longitudinal baseline, post and follow-up	Children	MRQ predicted mentees' increased active coping skills and support seeking.
10	Dallos & Carder-Gilbert	2018	Explore the experiences of young people and how these changed over a year of PROMISE mentoring in terms of attachment and coping skills.	UK	PROMISE	Diverse 6-20 months	Maltreated children	M=14 9-19	20	Mixed-methods	Children	Mentoring was experienced very positively, implying an increased sense of trust and reduced insecure attachment.
11	De Wit, DuBois, Erdem, Larose, & Lipman	2016	Examine the relationship between Children's and teens' mentoring status and outcomes	Canada	BBBS	Long-term 30 months	At-risk	6-17	859	Quasi-experimental Longitudinal Baseline/follow-up	Children	Mentees in a relationship lasting 12 or more months reported favorable outcomes. Re-matched girls displayed better outcomes than did never-mentored girls. Some evidence of negative outcomes for re-matched boys.
12	De Wit, DuBois, Erdem, Larose, & Lipman	2019	Examine predictors of mentoring relationship quality	Canada	BBBS	Long-term 30 months	At-risk	6-17	335 mentees, 356 parents	Quasi-experimental Longitudinal Baseline/follow-up	Children and parents	Predictors of RQ included minimal difficulties pairing mentees and mentors, perceptions of shared attributes, mentor emotional engagement and support, and

	Authors	Year	Aim	Country	Program	Duration Additional support	Population	Age (M/range)	Sample size	Design	Reporters	Main findings
												longer relationships.
13	De Wit, DuBois, Erdem, Larose, Lipman, & Spencer	2016	Examine patterns and correlates of early versus on-time mentoring closures	Canada	BBBS	Diverse, included matches that terminated before 12 months and others that lasted longer (up to 18 months when the survey was re-run)	At-risk	6-17	569	Quasi-experimental Longitudinal baseline/follow-up	Children	Early closures were positively associated with mentees' gender (girls), behavioral. difficulties, match difficulties, extrinsic reasons for joining the program. Early MR closures were negatively associated with mentees' perceptions of parents' emotional support, parental social support, high quality MR, weekly contact in MR, and parents' support of the MR.
14	De Wit, Wells, Elton-Marshall, & George	2017	Compare mentoring experiences and outcomes for Aboriginal (AB) and non-Aboriginal children and teens	Canada	BBBS	Diverse, due to early termination	At-risk	6-17	125 Aboriginal and 734 non-Aboriginal children and teens	Quasi-experimental Longitudinal baseline/follow-up	Children and teens	AB mentees were less likely than non-AB to be in a long-term continuous mentoring relationship.
15	DuBois, Herrera, & Higley	2018	Assess factors promoting the effectiveness and the length of the program	USA	Great Life Mentoring	Long-term 3 years on average (between 1 and15 years)	Outpatient mental health care preteens	Intervention M=9.92 (2.24) Controls M=10.15 (2.87)	66 mentees 66 controls	Quasi-experimental	Children	MR lasted longer when mentors had higher education, When the mentees did not have ADHD as a primary diagnosis and when the family had low but not very low income, mentees

	Authors	Year	Aim	Country	Program	Duration Additional support	Population	Age (M/range)	Sample size	Design	Reporters	Main findings
												showed better adaptive functioning in mental health care.
16	DuBois & Keller	2017	Assess the effectiveness of incorporating activities to promote thriving in the MR	USA	BBBS	Diverse 35.3% matches ended before 1 year	At-risk	M = 12.19 10-16	400 mentees in the treatment group and 406 in the matched control	RCT Longitudinal baseline/post/ follow-up	Teens	MR support increased mentees' thriving and in turn reduced problem behavior in the entire sample.
17	Erdem, DuBois, Larose, De Wit, & Lipman	2016	Explore indicators of positive youth development as mediators of associations of mentoring support with youth outcomes	Canada	BBBS	Diverse 66.5% long term, 33.5% terminated before one year.	At-risk	M=11.6 (2.13)	501	Quasi-experimental Longitudinal baseline/follow-up	Teens and parents	PYD mediated the association between mentoring support and decreased emotional and behavioral problems for active dyads.
18	Faith, Fiala, Cavell, & Hughes	2011	Explore mentors' changes throughout the mentoring	USA	CBM	Long-term 3 semesters	Aggressive elementary school students	Mentors M=20.2 (4.02) Children M=7.87 (1.04)	102 mentors	Quasi-experimental Longitudinal baseline/post	Mentors and children	Mentors who rated the MR as supportive tended to experience increased openness, conscientiousness, and agreeableness and less attachment avoidance.
19	Ferro, Wells, Speechley, Lipman, & De Wit	2014	Scale development	Canada	BBBS	Diverse	At-risk children and teens	6-17	491 children, 554 parents, 272 mentors	Quasi-experimental Longitudinal 2 point follow-up	Mentors, parents and children	Established convergent validity, good external and predictive validity. Mentor- and parent-reported MRQ

	Authors	Year	Aim	Country	Program	Duration Additional support	Population	Age (M/range)	Sample size	Design	Reporters	Main findings
												predicted mentoring status.
20	Gaddis	2012	Assess the contribution of the amount time spent, racial and SES similarity, and level of trust on mentees' outcomes	USA	BBBS	Short-term M =323.26 days	At-risk	M=12.3	355 mentees	Quasi-experimental Longitudinal baseline/post	Teens	Both the amount of time spent in a relationship and level of trust had positive effects on mentees.
21	Gettings & Wilson	2014	Examine mentors' commitment and relational maintenance	USA	Four different mentoring programs (3 CBM)	Diverse range: 1 month - 9 years M = 17.64 months (19.33)	At-risk	M=30.17 (13.86)	145 mentors	Cross-sectional	Mentors	Highly committed mentors were likely to employ relational maintenance strategies to maintain their desired relational balance
22	Goldner	2016	Examine the contribution of mentees' personality traits to MRQ and outcomes	Israel	PERACH	Short-term 8 months	At-risk	M= 9.58 (1.12)	167 mentees, 166 parents' 99 teachers	Quasi-experimental Longitudinal baseline/post	Children, parents and teachers	Mentees' positive personality traits correlated with mentees' expectations of mentoring. Agreeableness positively correlated with the quality of the relationship
23	Goldner & Mayseless	2009	Examine the contribution of MRQ to mentees' outcomes and the perceived contribution of the mentoring	Israel	PERACH	Short-term 8 months	At-risk	M=10.75 (1.07)	84 mentees, 78 mentors, 81 teachers	Quasi-experimental Longitudinal baseline/post	Children, mentors and teachers	Closeness in MR relationship predicted mentees' adjustment, improved functioning and mentoring perceived contribution.
24	Goldner & Scharf	2014	Examine the contribution of mentees' attachment dimension the	Israel	PERACH	Short-term 8 months	At-risk	M= 9.6 (1.1)	167 mentees, 151 mentors,	Quasi-experimental Longitudinal baseline/post	Children, mentors and teachers	Securely attached mentees forged close relationships with their mentors. A

	Authors	Year	Aim	Country	Program	Duration Additional support	Population	Age (M/range)	Sample size	Design	Reporters	Main findings
			MRQ and outcomes						157 teachers			positive MR was associated with mentees' adjustment at the end of mentoring.
25	Grossman & Rhodes	2002	Examine the impact of mentoring length on mentoring outcomes	USA	BBBS	Long-term M =12.9 months	At-risk	M=12.25	959; 487 treatment, 472 controls	RCT Longitudinal baseline/follow-up	Children	Mentees in relationships that lasted a year or longer reported the largest number of improvements. Mentees in relationships that terminated within a very short period of time reported decrements in functioning.
26	Herrera, DuBois, Grossman	2013	Assess mentees' risk and its effects on the MRQ and mentoring outcomes	USA	7 programs including 5 agencies of BBSS, a university-based program, and a program that targets young people in the community whose parents are in prison or have been in the correctional system.	Short-term M = 9.6 months	At-risk/low SES	M=11.39	1,310	RCT and a quasi-experimental study. Longitudinal baseline/follow-up	Pre-teens, mentors and parents	MR was associated with a reduction in depressive symptoms, increased social acceptance and academic achievements. Mentees with mentors who had substantial training reported higher quality and longer-lasting MR.
27	Herrera, Sipe, & McClanahan	2000	Explore mentors' experiences and the development of relationships in community-based programs and school-based programs	USA	98 mentoring CBM and SBM programs	Diverse, varied between programs	At-risk	22-49	1101 mentors	Cross-sectional	Mentors	Mentors in SBM spent more time on academics with mentees. More CBM mentors reported feeling very close to their mentees.

	Authors	Year	Aim	Country	Program	Duration Additional support	Population	Age (M/range)	Sample size	Design	Reporters	Main findings
28	Keller & DuBois, 2019	2019	Examine the contribution of program staff to supporting the development of MRQ	USA	BBBS	Long term- 15 months at assessment time.	At-risk	Teens M =12.16 Staff= M=31.73	450 Children 76 staff members, including 18 program super-visors	Quasi-experimental Longitudinal, Baseline/follow-up.	Teens , mentors and supervisors	Staff-level factors were associated with mentee-reported MR quality above and beyond individual mentor or mentee variables. Variability in MR quality was partially explained by level of work engagements, approaches to match support and supervisors' ratings of mentors' competence.
29	Kern, Harrison, Custer, & Mehta	2019	Assess to what extent mentor and mentee perceptions of the MRQ were influenced by age, race/ethnicity match, gender and specific topics discussed	USA	Check & Connect	Short-term 1 school year	At-risk	Mentees aged 15-18 Mentors aged 21-65	166 mentees 93 mentors	Cross-sectional (part of a larger RTC study)	Teens and mentors (mostly teachers)	Mentees reported the MR had a higher quality than the mentors perceived their mentees would. Most frequently discussed topics that were perceived helpful by both mentors and mentees were school and future plans.
30	Kupersmidt, Stump, Stelter, & Rhodes	2017	Identify indicators of premature match closure	USA	304 mentoring programs (92% one-to one, 56% CBM)	Diverse	At-risk	Mentees aged 6-19 Mentors aged 18-65	6468 mentees 6965 mentors	Cross-sectional	Children /teens and mentors	Age of mentees when matched and the cumulative risk index score predicted premature MR closure (family risk and risky health behaviors were most strongly associated with

	Authors	Year	Aim	Country	Program	Duration Additional support	Population	Age (M/range)	Sample size	Design	Reporters	Main findings
												early termination).
31	Langhout, Rhodes &, Osborne	2004	Evaluate differential influence of MR qualities on mentees' outcomes	USA	BBBS	Long-term (18 months)	At-risk	M=12.25 (1.71)	1138	RCT Longitudinal Baseline/follow-up	Teens	Mentees who described MR as providing moderate levels of activity, structure and conditional support derived the most benefit from the MR.
32	Lau, Zhou, & Lai	2017	Assess the contribution of MRQ to mentees' future-planning style, and career goal setting	Hong-Kong	Child Development Fund (CDF)	Long-term	At-risk	Intervention M=13.66 (1.93) Control M=12.94 (1.77)	187 mentees 208 controls	Quasi-experimental longitudinal baseline/follow up Treatment/ Control groups	Teens	Mentees with high MR quality showed better future planning style and career goal-setting self-efficacy compared to controls and mentees with low MR quality.
33	Larose et al.	2018	Examine three potential contributions (additive, hierarchical compensatory, hierarchical conditional) of mentor support to mentees' academic adjustment, taking into account interactions with support from mothers and teachers	Canada	BBBS	Long-term M=20.18 months (SD = 8.6)	At-risk	M=9.8 (2.2)	427	Quasi-experimental Longitudinal 6 point assessments	Children	Mentor support predicted positive changes in the mentees' academic adjustment mainly when mentees already reported high support from their mothers.
34	Larose, Savoie, De Wit, Lipman, & DuBois	2015	Assess the contribution of various types of activity to the quality of the MT	Canada	BBBS	Diverse (3-18 months)	At-risk	M=10.30 (2.16)	605	Quasi-experimental Longitudinal 3 point assessments	Children	Activity frequency was associated with perceptions of received support and relationship

	Authors	Year	Aim	Country	Program	Duration Additional support	Population	Age (M/range)	Sample size	Design	Reporters	Main findings
												quality. Higher frequency of recreational activities strengthened the association between perceived support and MR quality.
35	Lee, Krauss, Suandi, & Hamzah	2016	Understand MR processes and identify mentoring practices that enhance learning.	Malesia	Programme Remaja Perdana Rakan Muda (PRPRM), based on The Duke of Edinburgh's International Award Association program.	Long-term (minimum 12 months)	Adolescents and young adults	M =17.30 (.46) 14-25	90	Cross-sectional	Teens and young adults	Coaching contributed most to skill acquisition and knowledge learning, while facilitation contributed to attitude learning.
36	Liang, Tracy, Kenny, Brogan & Gatha	2010	Scale development for relational health indices for teens (RHI-Y).	USA		Not specified	133 first year middle school and 55 first-year high school students from both urban and suburban schools	13-18	188	Cross-sectional	Teens	Boys rated their MR lower in health compared to girls. MR was associated with social support, school engagement and reduced perceived stress.
37	Madia & Liutz	2004	Examine the contribution of interpersonal attraction to MRQ and intention to remain in the program	USA	BBBS	Short-term (less than 1 year) Range= 1-11 months M= 5.53 (2.20)	At-risk	Mentors: M = 30.80 (8.62) 19-59 Mentees: M=11.38 (2.50) 6-19	95	Cross-sectional	Mentors	Perceived similarity in extroversion and discrepancy between mentors' ideal versus actual roles predicted mentors' intentions to remain in the relationship. MR quality and interpersonal attraction mediated these associations.

	Authors	Year	Aim	Country	Program	Duration Additional support	Population	Age (M/range)	Sample size	Design	Reporters	Main findings
38	Marshall, Davis, Lawrence, Peugh, & Toland	2016	Scale development of the Mentors' Perceived Program Support Scale (MPPSS)	USA	19 programs (12 CBM)	Diverse	At-risk	M=36 (16.5) 18-79	664 mentors	Cross sectional	Mentors	Mentors' perceived program support was positively associated with strength of relationship, and mentors' age.
39	Martin & Sifers	2012	Identify the factors that promote mentors' satisfaction with MR	USA	BBBS	Not specified	At-risk	M = 30.34 (12.94) 19-70	81 mentors	Cross-sectional	Mentors	Perceived training and confidence predicted mentor satisfaction with MR.
40	Miller, Barnes, Miller, & McKinnon	2012	Identify how between-program differences impact success rates for mentored young people referred from the juvenile justice system	USA	A cluster-sampling of all 50 states	Not specified	At-risk mentees referred from the juvenile justice system	Not listed	1,197 Staff members	Cross-sectional	Mentors	More frequent interaction, longer relationships and mentors' formal training were associated with better outcomes.
41	Ng, Lai, & Chan	2014	Examine the impact of MRQ on mentees' psychosocial and educational development	Hong-Kong	MnM project -a community-based mentorship program	Short-term (maximum 1 years)	At-risk	M = 8.84 (1.04) 7-12	75 mentees 75 controls	Quasi-experimental. longitudinal: Baseline and 2 follow-ups assessments.	Children	Program participation was associated with greater academic pursuits and English competence.
42	Parra, DuBois, Neville, Pugh-Lilly, & Povinelli	2002	Identify mentoring processes	USA	BBBS	Short-term (maximum 1 years)	At-risk	Mentees M=10.09 (2.22) 7-14 Mentors M = 26.72 (7.77) 18-56	50 mentees 50 mentors	Quasi-experimental, longitudinal. Data collected monthly over one year.	Children and mentors	Mentors' ratings of their own efficacy predicted more contact and positive experiences in the MR. Feelings of closeness from both sides mediated the link between contact and MR benefits.

	Authors	Year	Aim	Country	Program	Duration Additional support	Population	Age (M/range)	Sample size	Design	Reporters	Main findings
43	Pedersen, Woolum, Gagne, & Coleman	2009	Examine objective and subjective data about mentoring relationship components	USA	Mentor Duluth, Minnesota	Long-term	At-risk	Mentees 4-18 Mentors 18-78	639 mentees	Mixed methods. Longitudinal – following data gathered for 10 years.	Children/ Teens	Inconsistent findings in the literature are explained by variability in MR (long wait for a match, multiple matches, differences in meeting frequencies).
44	Preston & Raposa	2019	Explore whether mentor characteristics influence the quality and impact of the mentor-mentee relationship	USA	College Mentors for Kids	Short-term (1 academic year)	at-risk	Mentees grades 3 through 6 Mentors M = 19 (1.00)	340 mentees and their mentors	Quasi-experimental Longitudinal baseline/post	Children and mentors	Mentors' depressive symptoms predicted negative perceptions of MR. Mentors' childhood stress predicted more positive perception of MR.
45	Raposa, Ben-Eliyahu, Olsho, L & Rhodes	2019	Test whether mentor-mentee similarities at baseline was indicative of longer-lasting mentoring relationships	USA	BBBS	Long-term (most matches lasted longer than 1 year)	At-risk	Mentees M = 10.60 (2.2.) 6-18 Mentors M = 29.1 (9.1)	9803 matches of mentor-youth	Quasi-experimental Longitudinal baseline/follow-up	Children and mentors	Similar race and ethnicity predicted longer MR. A shared dislike of activities was also associated with longer MR.
46	Rhodes, Grossman, & Resch	2000	Test pathways through which mentoring relationships influence academic adjustment	UAS	BBBS	Diverse	At-risk	M =12.25 10-16	959 mentees 487 treatment and 472 controls	RCT Longitudinal baseline/follow-up	Teens	MR was associated with better academic outcomes. This association was mediated by improvement in parental relationships
47	Rhodes, Lowe, Litchfield, & Walsh-Samp	2008	Explore the role of gender in shaping the course and quality of MR	USA	BBBS	Diverse	At-risk	M = 12.25 10-14	959 mentees 487 treatments 472 controls	RCT Longitudinal baseline/follow-up	Teens	Girls' MR lasted longer than boys MR. Girls were less satisfied than boys in shorter MR. Girls in longer MR reported the MR to be more helpful

	Authors	Year	Aim	Country	Program	Duration Additional support	Population	Age (M/range)	Sample size	Design	Reporters	Main findings
												compared to both boys and girls in shorter MR.
48	Rhodes, Reddy, & Grossman, 2005	2005	Test pathways through which mentoring relationships influence substance use	USA	BBBS	Diverse	At-risk	M = 12.25	928 mentees 456 treatments 472 controls	RCT Longitudinal baseline/follow-up	Teens	MR that lasted over 12 months predicted less substance use both directly and through improvement in mentees' self-perception and parental relationships.
49	Rhodes, Reddy, Grossman, & Lee	2002	Explore the role of race in shaping the course and quality of MR	USA	BBBS	Diverse	At-risk	M = 12.22	476 mentees 125 placed in cross-race matches 65 placed in same-race matches 286 controls	RCT Quasi-experimental Longitudinal baseline/follow-up	Teens	Minority mentees were less likely to report initiating alcohol when placed in a cross-race match. Minority boys in same-race matches reported less decline in scholastic competence and self-worth while minority girls in same-race matches reported less decline in school value and self-worth. Differences only emerged when considering both race and gender.
50	Rhodes, Reddy, Roffman, & Grossman	2005	Scale development – teen mentoring relationship quality inventory	USA	BBBS	Diverse	At-risk	M=12.10 5-18	347	Quasi-experimental Longitudinal baseline/follow-up	Children/ Teens	Positive MR was associated with length of match, MR was predictive of improved scholastic competence, school value and self-esteem.

	Authors	Year	Aim	Country	Program	Duration Additional support	Population	Age (M/range)	Sample size	Design	Reporters	Main findings
51	Rhodes, Schwartz, Willis, & Wu	2017	Scale development – mentor reported Strength of Relationship scale (SoR)	USA	BBBS	Long-term (min 1 year)	At-risk	Mentees M = 11.5 (2.4) 5-21 Mentors M = 32.2 (12) 17-83	5222 dyads	Quasi-experimental Longitudinal baseline/follow-up	Children/ teens and mentors	Mentors' and mentees' reports of positive affect were associated with MR longevity. Mentees' younger age, mentor's older age and mentor's higher education were associated with MR longevity beyond variance accounted for by the SoR.
52	Sale, Bellamy, Springer, & Wang	2008	Examine the contribution of MRQ to prevention objectives and social skills	USA	7 programs funded under a Youth Mentoring Initiative by the Center for Substance Abuse Prevention in the Substance Abuse and Mental Health Services Administration	Short-term (26-47 weeks)	At-risk	M = 11.9 8-18	1165 mentees 603 experi-ment and 562 control	RCT, Quasi-experimental Longitudinal baseline/post	Children /teens	Mentees who reported higher levels of trust, mutuality and empathy in MR showed more improvement in social skills compared to mentees who reported low quality MR.
53	Sánchez, Pryce, Silverthorn, Deane, & Dubois	2019	Examine the role of cultural mistrust and perceived support for ethnic-racial identity in a sample of girls of color.	USA	BBBS	Short-term 1 year	At-risk girls of color	M=11.75 (1.26)	40, 20 mentoring as usual, 20 mentoring plus enhancem ents	RCT, Quasi-experimental Longitudinal baseline/post	Teens	Mentor support for ethnic-racial identity was associated with increases in relational satisfaction and in identity exploration.
54	Schwartz et al.,	2014	Examine the impact of digital communication use on MRQ	USA, Canada	BBBS	Long-term M=20.6 months (21.8)	At-risk	M=35.1 (13.0) 18-79	258 mentors 147 staff program	Mixed methods	Mentors and staff	Digital media use between mentors and mentees was associated with greater MR quality and duration.

	Authors	Year	Aim	Country	Program	Duration Additional support	Population	Age (M/range)	Sample size	Design	Reporters	Main findings
55	Shamblen, Courser, Schweinhart, & Thompson	2019	Assess the impact of parental perceptions of mentor-mentee match on length and strength of MR	USA	BBBS (66% CBM)	Long-term M=16 months range: 1-35 months	At-risk	Mentees M=11.70 Mentors M=30.84 Parents 21-65	350 mentors 350 mentees 350 parents	Quasi-experimental Longitudinal baseline/post	Mentors, mentees and parents of mentees	Parental dissatisfaction with match predicted higher likelihood of match closure. Parental satisfaction with match predicted match strength.
56	Shlafer, Poehlmann, Coffino, & Hanneman	2009	Understanding the mentoring process of children of incarcerated parents	USA	BBBS	Short-term	Children of incarcerated parents	Mentees M=9 (3) 4-15 Mentors M=38 (16) 18-77 Caregivers (not specified)	57 dyads of mentees and mentors 252 interviews with mentors, 184 interviews with children, 184 interviews with caregivers	Mixed methods	Children Mentors Caregivers	Children who remained in MR longer than 6 months and met with mentors more frequently exhibited fewer internalizing and externalizing symptoms.
57	Silke, Brady, & Dolan	2009	Understand relational dynamics in formal adolescent mentoring programs	Ireland	BBBS	Long-term	At-risk	Mentees M =12.24 (1.27) 10-15 Mentors M = 30.98 (8.37) 18-56	76 mentees 76 mentors	Quasi-experimental Longitudinal 4-point assessments	Teens and mentors	Both mentors' and mentees' match satisfaction were associated with mentees' developmental outcomes and parental trust and social support.
58	Silverstein	2012	Explore how mentors' characteristics contribute to the mentoring outcomes	USA	Citywide mentoring program	Short-term (1 academic year)	At-risk (low income and minority back-ground)	Mentees 65.5% 6th grade, 34.5% 7th grade Mentors 18-over 61	119 mentor-mentee dyads	Retrospective Cross-sectional	Children and mentors	Mentors' prior experience with children and program content were associated with higher quality MR and with mentees' outcomes..

	Authors	Year	Aim	Country	Program	Duration Additional support	Population	Age (M/range)	Sample size	Design	Reporters	Main findings
59	Spencer, Drew, Walsh, & Kanchewa	2018	Examine the role of gender in MR duration and quality	USA	BBBS	3-6 months at time of survey)	At-risk	Mentees M = 12.32 (1.41) 9-15 Mentors M = 27.60 (7.02) 20-55 Guardians M = 39.72 (7.98) 27-64	67 triads of mentors, mentees and guardians	Mixed methods	Mentors, mentees and their guardians	Male mentors and mentees reported higher MR quality. Male matches were more likely to last at least 1 year. Female mentors were more often looking for a close relationship to develop quickly which resulted in a disconnection between their and their female mentees' expectations.
60	Spencer et al.	2019b	Study early MR endings	USA	BBBS	Short-term (match closed within 12 months)	At-risk	Mentees M =11.4 (1.8) 8-16 Parents M = 40.7 (9.7) 24-71 Mentors M = 32.2 (10.9) 18-76 Staff M = 32 (8.4) 22-58	72 Mentees 95 Mentors 104 Parents 124 Staff members	Mixed methods	Mentors, children, parents/ guardians and program staff	Most relationship endings were initiated by mentors. Most endings were unclear and confusing to program participants.
61	Stelter, Kupersmidt, & Stump	2018	Study predictors for match longevity of mentees in foster care	USA	BBBS – 75.48% community based, 24.52% school- or site-based	Long-term M = 13.31 months	Children and adolescents in foster care	Mentees 6-19 Mentors M = 32.31 (13.04) Staff M = 41.78 (10.48)	208 mentee – mentor dyads and staff members	Quasi-experimental Longitudinal baseline/follow-up Mentees in foster care were matched with mentees not in foster-care for analysis.	Children/ teens, mentors and staff	Mentees in foster care had shorter matches and matches were more likely to end prematurely.

	Authors	Year	Aim	Country	Program	Duration Additional support	Population	Age (M/range)	Sample size	Design	Reporters	Main findings
62	Stump, Kupersmidt, Stelter, & Rhodes	2018	Study the impact of mentoring and predictors for match longevity for Children of Incarcerated Parents (COIP)	USA	BBBS 55.05% community-based, 44.95% school- or site-based settings	Short-term M= 11.85 months	Children of incarcerated parents	Mentees M=11.59 Mentors M= 30.77 Staff M= 42 24-65	25,252: 10,579 matches including 2207 COIP matches	Mixed-methods Quasi-experimental Longitudinal baseline/follow up	Children, mentors, management staff	Having specific program goals, providing mentors with training, and receiving additional funding were associated with better outcomes for COIP matches such as longer and stronger matches.
63	Suffrin	2014	Study the role of social environments on mentor outcomes	USA	Chicago-based non-profit mentoring community initiative	Long-term M = 3.36 (3.29) years	At-risk	Mentees M= 12.52 Mentors' age was not specified	152 mentors	Cross-sectional	Mentors	Mentors' relationship with mentee's family, cultural competence and lower internalized White guilt were associated with positive outcomes.
64	Suffrin, Todd, & Sánchez	2016	Identify predictors of mentors' satisfaction	USA	72 mentoring organizations	Long-term M= 3.28 years	Not specified	Mentees M = 12.58 (3.56) Mentors' age was not specified	247 mentors	Cross-sectional	Mentors	Relationships with the mentee, mentee's family and mentoring organization predicted independent and interactive mentor satisfaction.
65	Thomson & Zand	2010	Scale development to examine how MR quality predicts other relationship-based outcomes	USA	Project: Youth Connect (PYC) focused on prevention, reduction and delay of substance abuse in at-risk youth	Short-term M = 6.8 months	At-risk	M=12.07 (1.56) 9-16	205	Quasi-experimental Longitudinal 2-point assessments	Teens	The quality of MR predicted mentees' scores in relationship-based outcomes (friendship and self-disclosure with adults)
66	Tierney, Grossman, & Resch	2000	Impact study of BBBS	USA	BBBS	Short-term M = 11.4 months	At-risk	10-16	959 487 treatment 472 controls	RCT Longitudinal baseline/follow up	Teens	Intervention mentees were less likely to use alcohol and drugs and to hit someone and had improved

	Authors	Year	Aim	Country	Program	Duration Additional support	Population	Age (M/range)	Sample size	Design	Reporters	Main findings
												academic and social outcomes.
67	Weiler, Boat, & Haddock	2019	Examining the role of mentoring experience and mentee risk in predicting the MRQ	USA	Campus Connections	Short-term 12 weeks	At-risk	Mentees M=14.3 (1.81) 11-18 Mentors M = 20.81 (2.94)	455 dyads of mentees and their mentors	Quasi-experimental Longitudinal Baseline and three time points	Teens and mentors	Environmental risk was negatively associated with MR quality, above and beyond adolescent risk. Mentors' perception of program structure, supportive MR and opportunities for skill building attenuated this association.
68	Weiler, Chesmore, Pryce, Haddock, & Rhodes	2019	Examine the relationship between mentor attunement and academic outcomes	USA	Campus Connections	Short-term 12 weeks	At-risk	Mentees M=14.9 (1.9) 11-18 Mentors M = 20.73 (3.46) 17-50	204 dyads of mentees and mentors	Quasi-experimental Longitudinal Baseline/ post	Teens and mentors	Mentees with attuned mentors reported better academic scores at post intervention, compared with mentees with mis-attuned mentors.
69	Williamson, Lawrence, Lyons, & Deutsch	2019	Examine the link between mentees' relationship characteristics and MR satisfaction	USA	Young Women Leaders Program – School-based program	Short-term (1 academic year)	At-risk girls	M=12.2 11-14	205	Quasi-experimental Longitudinal baseline/post	Preteens and Teens	Less maternal quality communication and trust was associated with lower quality MR. Higher maternal alienation was associated with high quality MR.
70	Zand et al.	2009	Scale development to assess mentees' perceptions of MR and its association with mentee competency	USA	Mentoring programs Youth Connect (PYC)	Short-term M = 10.7 (9.4) months	At-risk	Study 1: mentees M=12.7 (2.6) 9-19 Mentors M = 42.6 (18.6) 21-80 Study 2: Mentees	Study 1: 276 Study 2: 219	Quasi-experimental Longitudinal baseline/follow-up	Children/ Teens and mentors	The Mentor-Youth Alliance Scale (MYAS) predicted mentee competence in family and social relationships and life skills. More competent mentees were younger, more

	Authors	Year	Aim	Country	Program	Duration Additional support	Population	Age (M/range)	Sample size	Design	Reporters	Main findings
								M=12.05 (1.56)				likely to be female and had higher quality MR.
71	Zegers, Schuengel, van IJzendoorn, & Janssens	2006	Examine attachment representation effects on MRQ	Nether-lands	Youth treatment institution	Diverse M = 14 months (9) range: 2-35 months	High-risk adolescent offenders in treatment institutions	Mentees' age on admission M=15.8 (1.3) 13-20	81 mentees 33 professional caregivers	Quasi-experimental Longitudinal baseline/follow-up	Teens and mentors	In a subgroup of 28 mentees remaining longer in facilities, more secure mentees were perceived by mentors as having an increasingly secure base and decreasing avoidance of contact. More secure mentors were perceived as available as a secure base.

Note: MR=mentoring relationship, RQ= relationship quality, MRQ=mentoring relationship quality. Short-term<12 months, Long-term ≥12 months
