# Supplementary material for: Unpacking Community-Based Youth Mentoring Relationships: An Integrative Review
Source: Int J Environ Res Public Health. 2021 May 25;18(11):5666. doi: 10.3390/ijerph18115666 (PMC8198211; doi:10.3390/ijerph18115666)
Supplement: Supplementary file 1 [file ijerph-18-05666-s001.zip › Reviews and MetaAnalysesTable 16.8.20 after ed.pdf]

Table S3

Description of Reviews and Meta-Analyses

	Authors	Year	Aim	Type of review			Type of mentoring						
				Meta-analysis	Integrative	Systematic	CBM	SBM	Peer/ cross	Academic/Vocational	Natural	E-mentoring	Group
1	Christensen et al.	2020	Examine the effectiveness of skills-based versus non-specific, relational approaches to mentoring outcomes in 48 studies	X			X	X					
2	Deutsch & Spencer	2009	Explore measurement issues in MRQ		X		X	X			X		
3	DuBois et al.	2002	Identify best-practices promoting the effectiveness of mentoring in 55 studies	X			X	X	X	X			
4	DuBois et al.	2011	Identify best-practices promoting the effectiveness of mentoring in 73 studies	X			X	X	X	X		X	X
5	Dutton	2018	Review the role of self-disclosure		X		X	X	X		X		
6	Eby et al.,	2013	Identify antecedents, correlates, and consequences of mentees' perceptions MR 73 studies	X			X			X			
7	Farruggia, Bullen, Solomon, Collins, & Dunphy	2017	Examine the cultural context in New Zealand			X	X		X			X	X
8	Jarjoura, DuBois, Shlafer, & Haight	2013	Summarize shared input during listening sessions to enhance the effectiveness of mentoring for children of incarcerated parents		X		X	X			X		
9	Raposa et al.	2019	Identify mentoring effect size and moderators in 70 studies	X			X	X					
10	Rhodes & DuBois	2006	Review scientific data on the topic of MRQ and program practices		X		X	X	X		X		
11	Schwartz, Lowe, & Rhodes	2012	Review mentoring contribution to mentees' competence		X		X	X	X		X		
12	Stewart, C., & Openshaw	2008	Explore the term mentors, mentoring outcomes and MRQ characteristics		X		X				X		
