# Supplementary figures and images for: Unpacking Community-Based Youth Mentoring Relationships: An Integrative Review
Source: Int J Environ Res Public Health. 2021 May 25;18(11):5666. doi: 10.3390/ijerph18115666 (PMC8198211; doi:10.3390/ijerph18115666)

Figure S1. *Flow Chart of Methodology Applied to Screen Records*

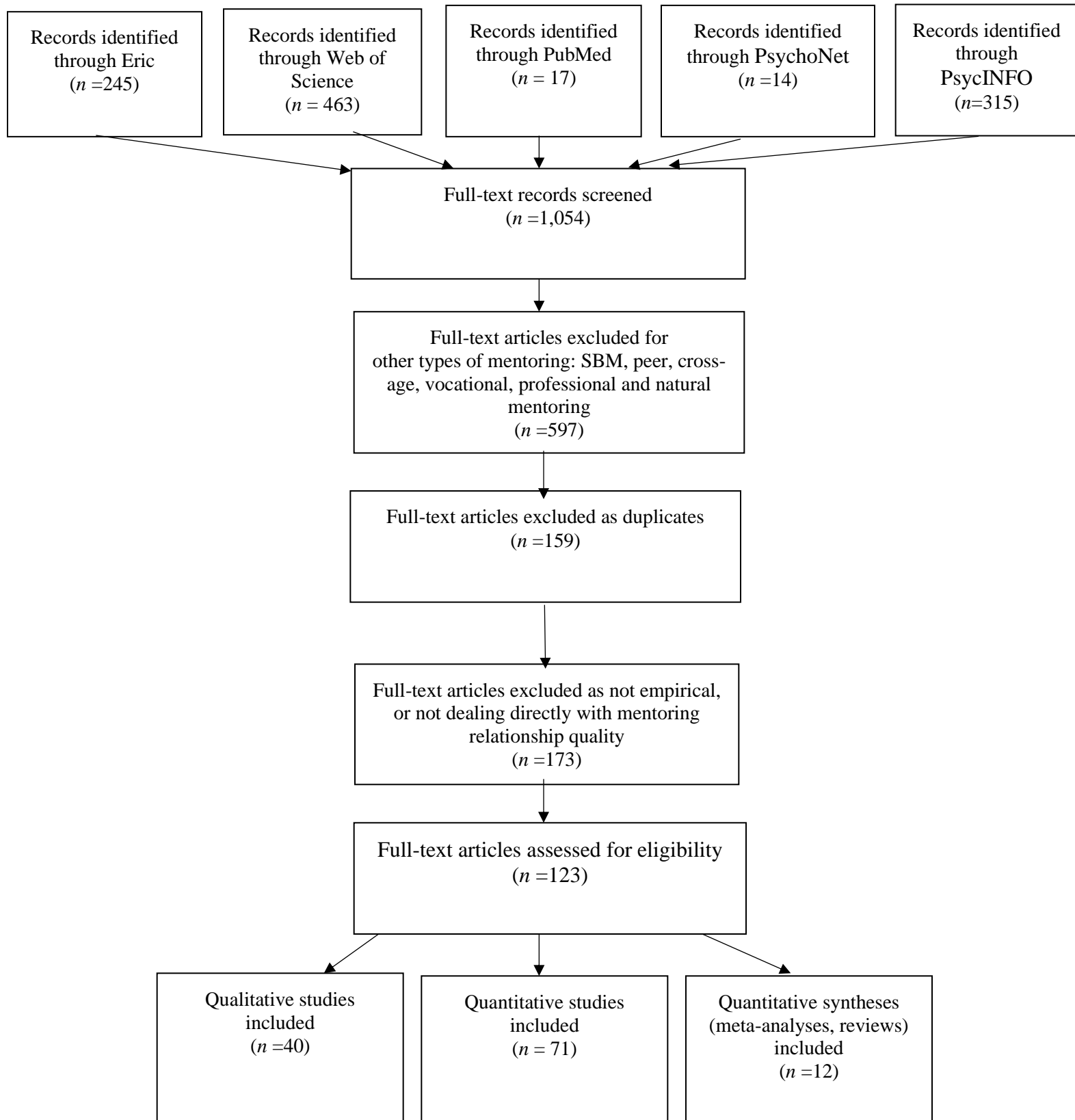

Supplement: Supplementary file 1 [file ijerph-18-05666-s001.zip › flow 6.2.21.pdf]
